# An autonomous TCR signal-sensing switch influences CD4/CD8 lineage choice in mice

**DOI:** 10.1038/s42003-022-02999-5

**Published:** 2022-01-21

**Authors:** Jayati Basu, Jikun Zha, Emmanuelle Nicolas, Michael Coulton, Philip Czyzewicz, Xiang Hua, Lu Ge, Dietmar J. Kappes

**Affiliations:** Fox Chase Cancer Center, 333 Cottman Avenue, Philadelphia, PA 19111 USA

**Keywords:** Lymphocyte differentiation, Immunogenetics

## Abstract

How multipotential cells initiate distinct gene expression programs in response to external cues to instruct cell fate choice remains a fundamental question in biology. Establishment of CD4 and CD8 T cell fates during thymocyte development is critically regulated by T cell receptor (TCR) signals, which in turn control expression of the CD4-determining transcription factor ThPOK. However, the mechanism whereby differential TCR signals are molecularly interpreted to promote or antagonize ThPOK expression, and thereby CD4 versus CD8 lineage fates remains unknown. Here we show, using reverse genetic and molecular approaches that an autonomous, position-independent TCR-sensing switch is embedded within the ThPOK locus. Further, using an in vivo mutagenesis approach, we demonstrate that differential TCR signals are interpreted during lineage commitment by relative binding of EGR, NFAT and Ebox factors to this bistable switch. Collectively our study reveals the central molecular mechanism whereby TCR signaling influences differential lineage choice. Ultimately, these findings may provide an important new tool for skewing T cell fate to treat cancer and autoimmune diseases.

## Abstract (web summary)

Basu Jayati et al. examine how T-cell receptor (TCR) engagement with MHC ligands can contribute toward adoption of CD4 and CD8 cell fates during development. By using an in vivo mutagenesis approach in mice, their results suggest potential mechanisms linking TCR signaling to ThPOK expression and differential immune lineage choice.

## Introduction

How bipotential cells use environmental cues to precisely orchestrate distinct gene expression programs in order to promote alternate developmental fates, remains a fundamental question in biology. CD4-CD8 T-cell lineage commitment in the thymus provides a valuable model system to understand this process^1^. T cells develop in the thymus from the early thymocyte progenitor stage to the mature SP CD4 and CD8 stages via a precisely ordered series of intermediate steps. The CD4+ CD8+ (double positive, or DP) stage is particularly important, as thymocytes first express a complete αβTCR at this stage, allowing them to engage MHC on antigen-presenting cells and undergo positive selection. Prior to positive selection DP thymocytes are believed to be lineage-uncommitted, i.e., not biased towards either CD4 or CD8 lineage choice. After positive selection, DP thymocytes split into alternate CD4 and CD8 lineages, by undergoing selective loss of one of the coreceptors. There is a tight correlation between a thymocyte’s TCR specificity for MHC class II or I, and differentiation to the CD4 or CD8 lineages, respectively.

The near-perfect correlation between lineage choice and MHC restriction can be explained by the kinetic-signaling model, which postulates that relatively long or short TCR signals promote CD4 versus CD8 commitment, respectively^2–4^. At the molecular level, it is established that the transcription factor (TF) ThPOK is necessary and sufficient to drive CD4 commitment, and to prevent CD8 commitment, of developing thymocytes^5,6^. Accordingly, ThPOK protein levels are higher in class II-restricted thymocytes than in class I-restricted thymocytes, suggesting a causal link between TCR engagement and ThPOK expression^7^. However, the fundamental question of how differential TCR signals control lineage-specific ThPOK expression, and thereby alternate lineage fate, remains to be resolved.

What is known so far is that ThPOK expression in thymocytes and mature T cells is controlled primarily at the transcriptional level via several stage- and lineage-specific cis elements^8,9^. Of particular importance is the 400 bp *ThPOK* silencer, Sil^ThPOK^, which is located 3 kb upstream of the distal *ThPOK* promoter^8,9^. Germline deletion of the Sil^ThPOK^ in mice causes promiscuous expression of ThPOK and diverts all thymocytes towards the CD4 lineage, demonstrating that the Sil^ThPOK^ is essential for repression of *ThPOK* transcription in cells that would normally adopt the CD8 lineage. Our understanding of how the Sil^ThPOK^ is regulated, however, remains rudimentary. Deletion of 2 Runx consensus binding motifs severely impairs silencing function^8,9^, and mice lacking Runx1 and Runx3 or the obligate Runx-binding partner Cbfb, exhibit loss of the T-cytotoxic lineage. Interestingly, while constitutive expression of ThPOK causes redirection of class I-restricted thymocytes to the CD4 lineage, overexpression of Runx3 is not sufficient to redirect MHC II-restricted thymocytes to the CD8 + lineage^10^. Furthermore, Runx factors are bound to the Sil^ThPOK^ at all stages of thymic development, indicating that differential binding by Runx factors is not responsible for differential silencer function in class I- versus II-restricted thymocytes. Hence, the molecular basis for how differential TCR signals regulate ThPOK expression in class I- versus class II-restricted thymocytes remains to be determined.

Regulation of the *Cd4* gene during thymic development somewhat parallels that of *ThPOK*, in that it is also controlled by a stage-specific silencer element, Sil^CD4^, which selectively represses *Cd4* transcription in SP CD8 but not SP CD4 thymocytes^11–13^, and which contains functionally critical Runx-binding sites^14,15^. However, no evidence has emerged to date that *Cd4* transcription and the Sil^CD4^, in particular, are regulated by TCR signals. Indeed, this would seem intuitively unlikely given that *Cd4* is transcribed in both unsignaled DP thymocytes and strongly signaled SP CD4 thymocytes.

As outlined above, the molecular genetic mechanisms by which TCR-dependent regulation of *ThPOK* transcription is controlled remain unknown. Here, we seek to resolve this critical issue using an in vivo gene targeting approach. First, through reciprocal swapping of the Sil^ThPOK^ with the Sil^CD4^, we provide molecular genetic proof that TCR signals directly target the Sil^ThPOK^. Second, using precise in vivo gene editing we identify an autonomous, position-independent TCR-sensing switch within the Sil^ThPOK^ that controls *ThPOK* expression in developing thymocytes. Collectively, our study defines the central molecular genetic mechanism whereby TCR signaling influences lineage choice via regulation of *ThPOK* expression.

## Results

### In vivo silencer swap reveals the autonomous and position-independent function of Sil^ThPOK^

While we previously showed that strong TCR signals induce *ThPOK* transcription in thymocytes^8^, the molecular mechanisms that connect TCR signals with *ThPOK* induction are unknown. We reasoned that TCR signals may induce *ThPOK* expression either by (1) activation of positive regulatory elements (enhancers/promoters), or (2) inactivation of the Sil^ThPOK^ silencer (Supplementary Fig. 1). To genetically test whether the Sil^ThPOK^ encodes the autonomous and locus-independent capacity to sense differences in MHC class I- versus class II-restricted TCR signaling, we generated Sil^ThPOK^ swap mice, in which the Sil^ThPOK^ is inserted into the *Cd4* gene in place of its own Sil^CD4^ silencer element (CD4^ThPOKsil^ mice). We used this approach because Sil^CD4^ and Sil^ThPOK^ share important functional attributes, i.e., both are active in developing class I-restricted thymocytes and both are dependent on binding of Runx factors^14,16^. The murine Sil^ThPOK^ and Sil^CD4^ elements are predicted to interact with 183 and 164 TFs, respectively, many of which are differentially regulated between mature CD4 and CD8 thymocytes and thus may contribute to lineage-specific regulation of the Sil^ThPOK^ and Sil^CD4^ elements. Interestingly, 129 of these TFs are predicted to bind both Sil^ThPOK^ and Sil^CD4^ elements (Supplementary Fig. 2a–c), suggesting substantial commonality in control of both elements. On the other hand, some TFs are predicted to show unique or highly preferred binding to the Sil^ThPOK^ versus Sil^CD4^ elements, suggesting a selective role in the control of the Sil^ThPOK^. Hence, we hypothesized that the Sil^ThPOK^ might substitute in many respects for the Sil^CD4^, but that some functions might be specialized, including the ability to respond to differential TCR signals.

**Fig. 1:**
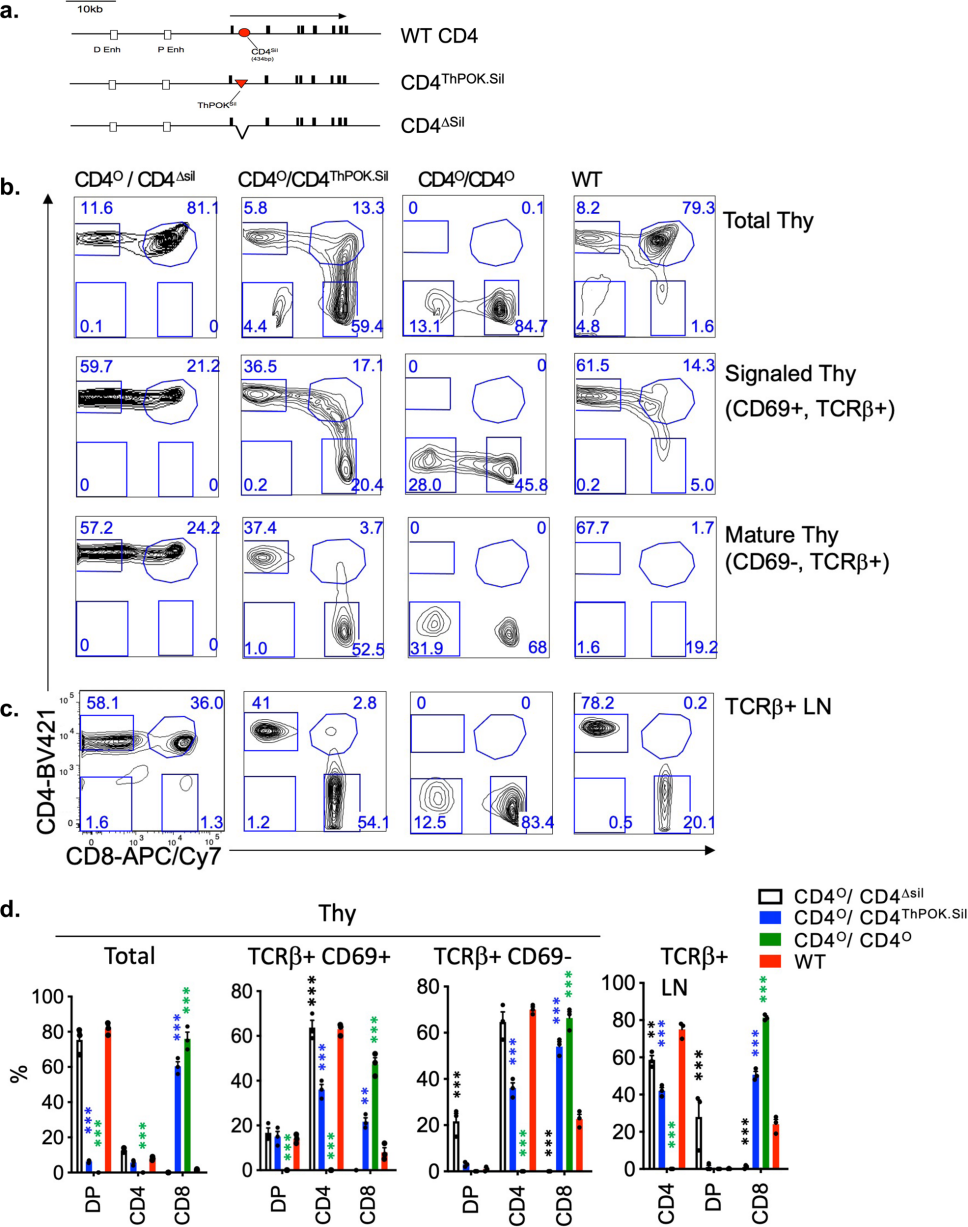
Sil^ThPOK^ represses gene expression at the DP stage in CD4^ThPOK.Sil^ knock-in mice. **a** Schematic of *Cd4* gene organization in wt mice (top row), CD4^ΔSil^ (second row) or CD4^ThPOK.Sil^ knock-in mice (bottom row). Black boxes indicate exons. Enhancers are shown as white boxes, the *Cd4* silencer as a red circle, and the *ThPOK* silencer as a red triangle. **b** FACS analysis of CD4, and CD8a expression of total thymocytes (top row), or indicated gated thymocyte subsets (bottom 2 rows) of CD4^Δsil/Δsil^, CD4^O/ThPOK.Sil^, CD4^O/O^, and wt mice. Note that CD4 expression is severely reduced at immature DP-like stage, but is substantially upmodulated in many CD69+ TCRβ+ thymocytes that have received a recent TCR signal. **c** FACS analysis of CD4 and CD8a expression of total mesenteric lymph node (LN) cells (top row), or gated TCRβ+ LN cells subsets (bottom row) of same strains of mice as above. Results are representative of three independent experiments (*n* = 3, for each strain). **d** Plots showing % of DP, SP CD4, SP CD8, and DN thymocytes for mice of indicated genotypes. *N* = 3 independent animals. Data are presented as mean values +/− SEM. A *P* value < 0.05 was considered significant. Significant differences were determined by one-way ANOVA with post hoc Tukey HSD (honest significant difference), and indicated by asterisks (*P < 0.01; **P < 0.005; ***P < 0.001). Statistical significance was calculated for each indicated mutant line relative to wt mice.

We performed the Sil^ThPOK^ > Sil^CD4^ swap in 2 steps: first we generated mice in which the Sil^CD4^ is deleted (CD4^Δsil^ mice) (Fig. 1a). As previously reported, CD4^Δsil^ mice exhibit gain of CD4 expression by mature CD8 T lymphocytes both in heterozygous and homozygous condition, consistent with the fact that the Sil^CD4^ is required to suppress *Cd4* expression after CD8 commitment^9^ (Fig. 1b–d). Next, we inserted the Sil^ThPOK^ into the CD4^Δsil^ allele (at the site of the Sil^CD4^ deletion) to test whether it was able to restore normal regulation of *Cd4* expression (CD4^ThPOKsil^ mice), as assessed at the single-cell level by FACS (Fig. 1a). Expression of the CD4^ThPOK.Sil^ allele was first assessed in heterozygous CD4^ThPOKsil/+^ mice, in which the CD4^ThPOK.Sil^ allele is expressed in combination with a normal *Cd4* allele regulated by the endogenous Sil^CD4^. T cells from these mice showed no change in coreceptor expression pattern in thymocytes or peripheral T cells, including no gain of CD4 expression on CD8 T cells, suggesting that the Sil^ThPOK^ substituted fully for the function of the endogenous Sil^CD4^ in the CD8 lineage and/or is functionally complemented by the wt *Cd4* allele. We next generated hemizygous CD4^ThPOKsil/o^ mice, in which only the CD4^ThPOKsil^ allele is expressed, by crossing CD4^ThPOKsil^ mice to *Cd4*-deficient (CD4^o/o^) mice^17^. Importantly, these mice show severe alteration of CD4 expression in the thymus, i.e., downregulation of CD4 on most immature (TCR^lo/−^) thymocytes. As a result, normal immature DP (CD4 + CD8 + TCR^lo^) thymocytes are largely replaced by aberrant SP CD8 TCR^lo^ cells (distinguishable from mature SP CD8 cells by the absence of surface TCR; Supplementary Fig. 3) (Fig. 1b–d). Thus, insertion of Sil^ThPOK^ into the *Cd4* locus represses transcription of *Cd4* in most DP thymocytes, revealing an inherent capacity of the Sil^ThPOK^ to repress transcription at the DP stage.

**Fig. 2:**
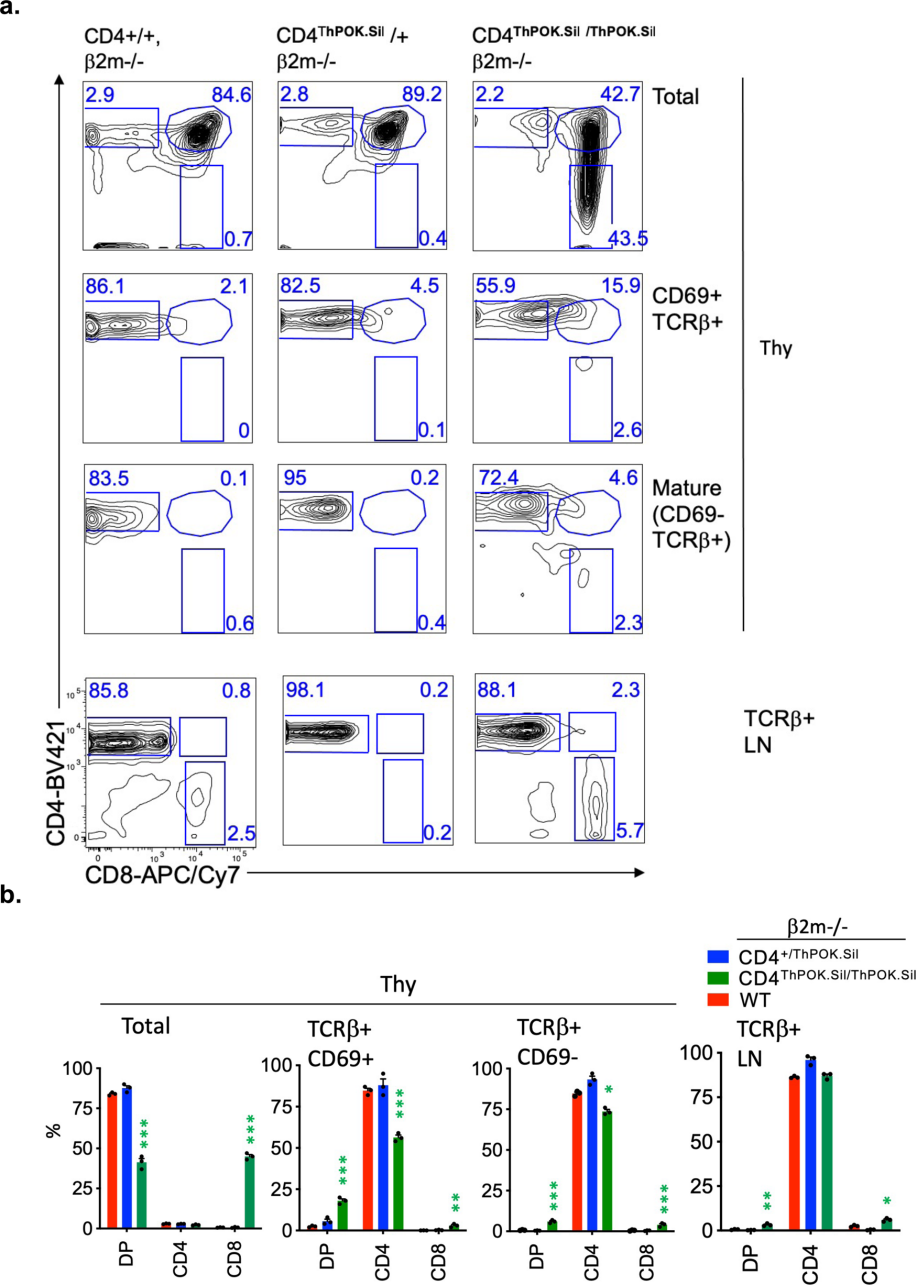
Sil^ThPOK^ causes partial redirection of MHC class II-restricted thymocytes to CD8 lineage in CD4^ThPOK.Sil^ knock-in mice. **a** FACS analysis of CD4 and CD8a expression by indicated thymic or peripheral lymphocyte populations of wt, CD4^ThPOK.Sil/+^ or CD4^ThPOK.Sil/ThPOK.Sil^ mice crossed to the β2 m-deficient background. **b** Plots showing % of DP, SP CD4, and SP CD8 thymocytes and LN for mice of indicated strains on β2m−/− background. *N* = 3 independent animals, for each strain. Data are presented as mean values +/− SEM. A *P* value < 0.05 was considered significant. Significant differences between indicated mutant mice and WT mice were determined by one-way ANOVA with post hoc Tukey HSD (honest significant difference), and indicated by asterisks (*P < 0.01; **P < 0.005; ***P < 0.001). Statistical significance was calculated for each indicated mutant line relative to β2m−/− mice.

Downmodulation of CD4 at the DP stage is expected to impair TCR signaling by MHC class II-restricted thymocytes leading to a defect in their differentiation, similar to CD4 knockout mice in which most class II-restricted thymocytes are redirected to the CD8 lineage^18^. Indeed, CD4^ThPOKsil^/CD4^o^ mice show a reduction in the frequency of SP CD4 thymocytes and peripheral CD4 T cells compared to wt mice (~50% of normal) (Fig. 1b–d). To distinguish whether this reflects a block in the development of class II-restricted cells, or redirection to the CD8 lineage, we backcrossed CD4^ThPOK.Sil^ mice to a β2m-deficient background, in which only MHC class II-restricted cells can develop^19^. While, the frequency of SP CD4 cells was reduced, particularly in the periphery (spleen and lymph node), the CD8 compartment was not substantially increased (Fig. 2a, b), implying that the defect in CD4 generation reflects a partial block in development rather than redirection to the CD8 lineage. We speculated that remaining CD4 cells in CD4^ThPOK.Sil/ThPOK.Sil^ mice might be strongly skewed toward higher affinity TCRs that are relatively less dependent on CD4 coengagement for efficient TCR signaling. To test this hypothesis, we introduced the AND TCR transgene, which recognizes the MHC II I-A^k^ allele with high affinity, onto the CD4^ThPOK.Sil^ background. Notably, AND TCR+ I-A^k^+ CD4^ThPOK.Sil/ThPOK.Sil^ mice showed normal frequencies of mature CD4 thymocytes, supporting the view that high-affinity TCRs are unaffected on the CD4^ThPOK.Sil^ background (Supplementary Fig. 4a; note that SP CD8 thymocytes are immature DP-like cells, not mature CD8 thymocytes). In contrast to most MHC class II-restricted cells, the development of class I-restricted T cells appears normal. Thus, on the MHC class II-deficient background, in which only class I-restricted cells can develop, all cells mature appropriately to the CD8 lineage and give rise to normal frequencies of CD8 cells (Supplementary Fig. 4b).

### Sil^ThPOK^ encodes inherent capacity to respond to TCR signal

To directly test whether the Sil^ThPOK^ encodes the inherent capacity for TCR responsiveness in the context of CD4^ThPOKsil^ mice, we treated CD4^ThPOKsil/ThPOKsil^ MHC II deficient mice in vivo with anti-TCR antibody, as an inducer of strong TCR signaling, and used *Cd4* transcription from the knock-in locus as a readout of TCR responsiveness. We focused on CD69+ CD4lo/− CD8+ thymocytes, because they are the functional equivalent of CD69+ DP thymocytes (since Sil^ThPOK^ is repressing *Cd4* expression at the immature DP equivalent stage). In normal thymic development the first signaled thymocytes appear as CD69+ CD4+ CD8+^20^. Importantly, we detected a strong increase of *Cd4* mRNA levels in activated (CD69+) thymocytes derived from treated versus untreated mice, providing direct biochemical evidence that the Sil^ThPOK^ encodes the inherent capacity for inducibility in an autonomous and position-independent manner in response to strong TCR signals (Supplementary Fig. 5a, b). Since TCR signaling increases *Cd4* expression from the CD4^ThPOK.Sil^ knock-in locus, TCR signaling appears to counteract Sil^ThPOK^ function. Altogether our data indicate that the Sil^ThPOK^ represses the *Cd4* locus at the DP stage via a cell-autonomous and position-independent mechanism, and confers susceptibility to inactivation by strong TCR signals in activated (CD69+) thymocytes.

### Sil^CD4^ element cannot substitute for the TCR-sensing capacity of the Sil^ThPOK^

**Fig. 3:**
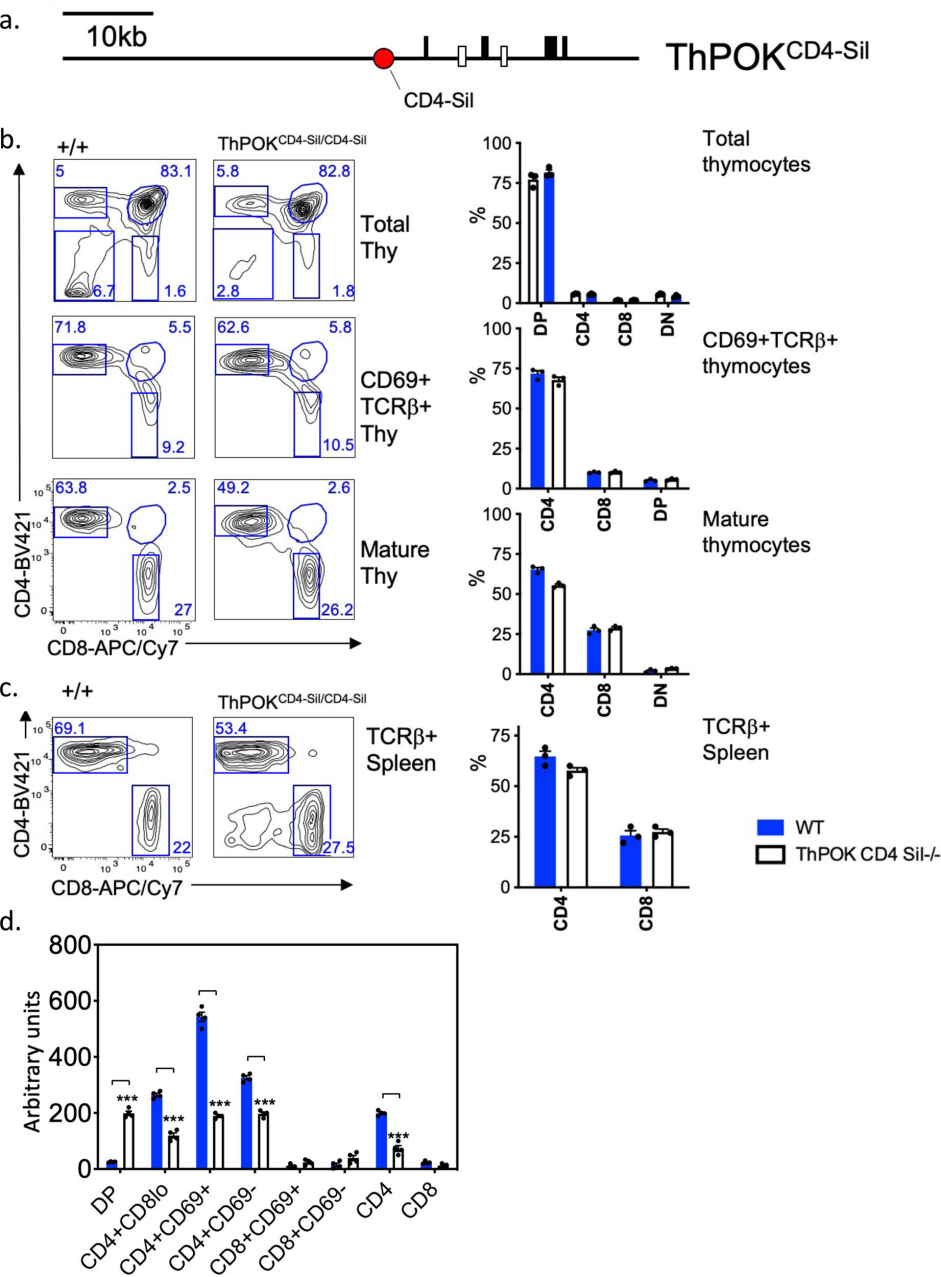
Substitution of the *Cd4* silencer for the *ThPOK* silencer in the context of the endogenous *ThPOK* locus maintains normal SP CD4 and CD8 frequencies. **a** Schematic of *ThPOK* gene organization in ThPOK^CD4.Sil^ knock-in mice. Black boxes indicate exons. Enhancers are shown as white boxes, and the *Cd4* silencer as a red circle. **b** FACS analysis of CD4 and CD8α expression by indicated thymic populations of wt or ThPOK^CD4-Sil/CD4-Sil^ mice. **c** FACS analysis of CD4 and CD8a expression by indicated splenic populations of same mice as in panel b. Graphic comparison of proportions of DN, SP CD4, and SP CD8 subsets within gated TCRβ + spleen lymphocytes of wt or ThPOK^CD4-Sil/CD4-Sil^ mice (*n* = 7) (right panel). There were no statistically significant differences between mice of ThPOK^CD4.Sil^/^CD4.Sil^ and ThPOK+/+ genotypes by one-way ANOVA with post hoc Tukey HSD. **d** RT-PCR analysis showing relative expression of *ThPOK* mRNA in indicated sorted thymocyte subsets of wt or ThPOK^CD4-Sil/CD4-Sil^ mice. Results are a combination of three replicates per strain. RT-PCR data represent four technical replicates, each derived from pooled RNA of three animals. Data are presented as mean values +/− SEM. A *P* value < 0.05 was considered significant. All FACS results are representative of at least three experiments. Statistical significance was determined between mice of ThPOK^CD4.Sil/CD4.Sil^ and ThPOK^+/+^ genotypes by one-way ANOVA with post hoc Tukey HSD, and indicated by asterisks (*P < 0.01; **P < 0.005; ***P < 0.001).

Next, we tested the proposition whether the capacity to respond to TCR signals is unique to the Sil^ThPOK^ or might be shared with other regulatory elements at the ThPOK locus. For this purpose, we carried out a converse silencer swap approach in which the Sil^ThPOK^ was replaced by an exogenous silencer element. We first used the human adult-specific γ-globin silencer^21^. However, this silencer failed to repress *ThPOK* expression in DP thymocytes or in any other thymocyte stage (Supplementary Fig. 6), suggesting that the γ-globin gene does not work in the context of T lymphocytes. To overcome this limitation, we instead used the Sil^CD4^, which is known to be active in the T-cell lineage. Accordingly, we replaced the 418 bp Sil^ThPOK^ by the 434 bp Sil^CD4^ in the context of the endogenous *ThPOK* locus, to generate ThPOK^CD4Sil^ mice (Fig. 3a). As mentioned above, the Sil^CD4^ is selectively active in SP CD8 cells, but unlike the Sil^ThPOK^ is not active in DP thymocytes. FACS analysis of homozygous ThPOK^CD4Sil^ knock-in mice showed essentially normal CD4:CD8 T-lymphocyte ratios in mature thymocytes and peripheral T cells, consistent with the efficient functional substitution for the Sil^ThPOK^ by the Sil^CD4^ during T-cell development (Fig. 3b, c). Furthermore, RT-PCR analysis showed normal repression of *ThPOK* transcription in sorted mature SP CD8 thymocytes, However, expression levels of *ThPOK* at other stages of T-lymphocyte development were severely altered, i.e., upmodulated in DP T-lymphocyte precursors, and downmodulated in CD4+ 8lo (eightfold) and SP CD4 lymphocytes in thymus and peripheral immune sites (five- to sixfold) (Fig. 3d).

**Fig. 4:**
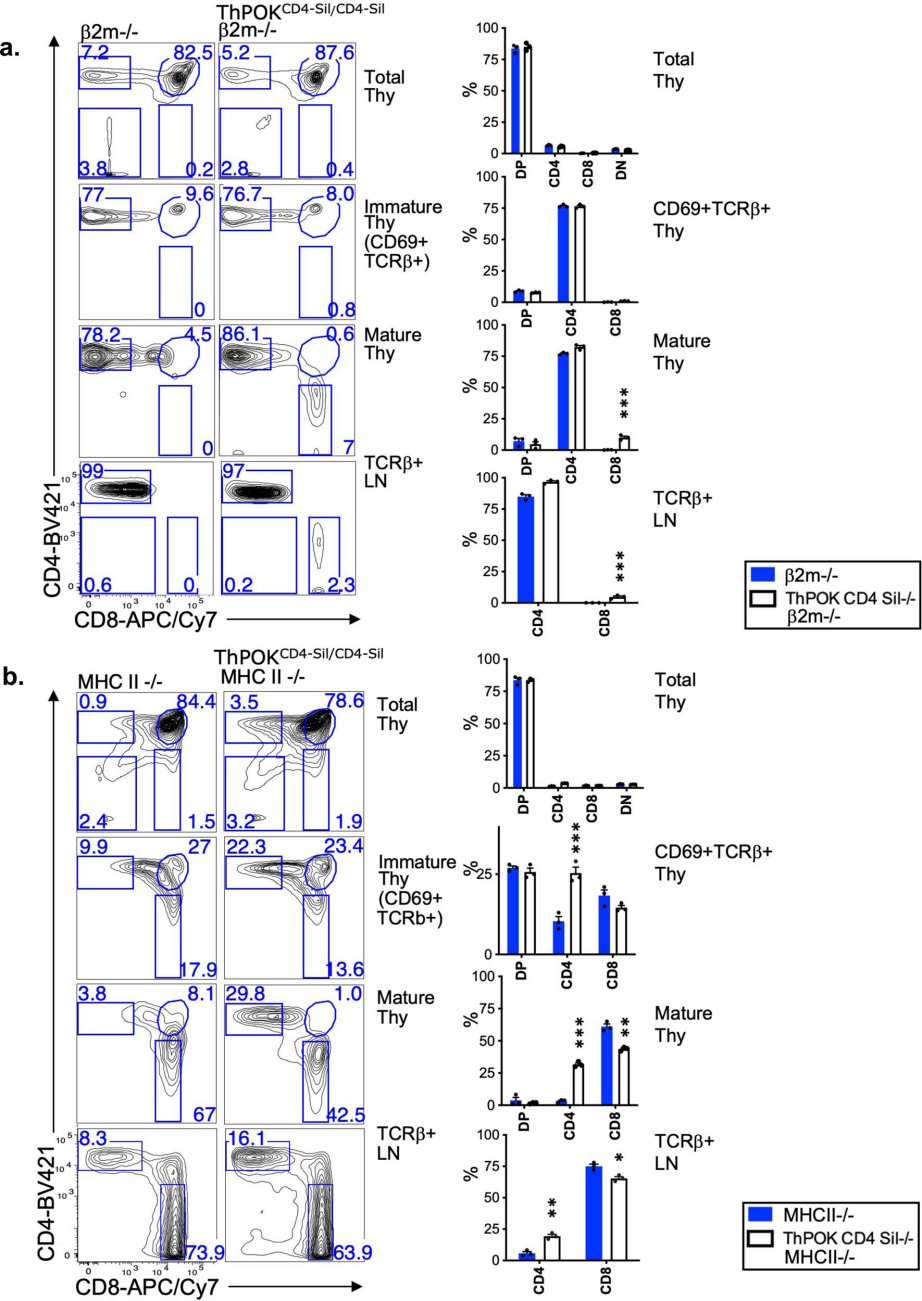
Substitution of the *Cd4* silencer for the *ThPOK* silencer perturbs the correlation between MHC restriction and lineage choice. **a** FACS analysis of CD4 and CD8a expression by indicated thymic or peripheral lymphocyte populations of wt or ThPOK^CD4-Sil/CD4-Sil^ mice crossed to the β2 m-deficient background. **b** FACS analysis of CD4 and CD8α expression by indicated thymic or peripheral lymphocyte populations of wt or ThPOK^CD4-Sil/CD4-Sil^ mice crossed to the MHC II-deficient background. *N* = 3 independent animals per strain. Data are presented as mean values +/− SEM. A *P* value < 0.05 was considered significant. Statistical significance was determined between mice of ThPOK^CD4.Sil/CD4.Sil^ and ThPOK^+/+^ genotypes on β2m−/− or MHC II−/− background by one-way ANOVA with post hoc Tukey HSD, and indicated by asterisks (*P < 0.01; **P < 0.005; ***P < 0.001). Statistical significance was calculated for each indicated mutant line relative to β2m−/− mice (**a**), or MHC II−/− mice (**b**).

Given the severe misregulation of *ThPOK* levels in ThPOK^CD4Sil^ mice, we questioned whether the coreceptor expression pattern of their SP thymocytes really correlates appropriately with MHC restriction, despite the superficially normal CD4:CD8 ratio. To definitively address this issue, we crossed ThPOK^CD4Sil^ mice with MHC class I- or II-deficient mice (or TCR transgenic mice expressing class II- or I-restricted TCRs) to restrict development exclusively to class II- or I-restricted cells, respectively. ThPOK^CD4Sil^ MHC I−/− mice developed a considerable proportion of misdirected class II-restricted CD8 T lymphocytes in the thymus compared to control MHC I−/− mice, of which a few are also detected in the periphery, although less than in the thymus, presumably reflecting homeostatic mechanisms that favor class II-restricted T lymphocytes expressing the appropriate CD4 coreceptor (Fig. 4a). Interestingly, redirected CD8 SP thymocytes were evident only at the mature CD69- stage, not among signaled (TCRβ + CD69+) thymocytes, which are thought to contain lineage-committed semi-mature CD4 and CD8 SP cells in WT mice. Hence, derepression of ThPOK appears to be influencing post-commitment events in CD8 SP differentiation. Even more strikingly, ThPOK^CD4Sil^ MHC II−/− mice develop almost equal frequencies of mature CD4 and CD8 T lymphocytes in the thymus, in contrast to MHC II−/− control mice (Fig. 4b). The proportion of SP CD4 cells was also elevated in the periphery compared to control MHC II−/− mice, although to a lesser degree (Fig. 4b). Conversely, there is substantial misdirection of class II-restricted thymocytes to the CD8 lineage in ThPOK^CD4Sil^ MHC I−/− mice compared to control MHC I−/− mice (Fig. 4b). A few misdirected MHC II-restricted CD8 T cells are also detected in the periphery, although proportionally less than in the thymus. These data demonstrate that the Sil^CD4^ cannot substitute functionally for the Sil^ThPOK^, especially failing to repress ThPOK transcription in DP thymocytes and causing inappropriate reduction of ThPOK transcription in CD4+ 8lo and SP CD4 thymocytes. Most importantly, other endogenous ThPOK regulatory elements cannot confer TCR sensitivity in the absence of the Sil^ThPOK^. As a consequence, thymocytes from ThPOK^CD4Sil^ mice exhibit random lineage commitment independent of MHC specificity (Fig. 4a, b). Altogether these results suggest that *ThPOK* promoters and enhancers are insufficient to support normal levels of *ThPOK* transcription in response to TCR signal when linked to the Sil^CD4^. Thus TCR sensitivity is exclusive to the Sil^ThPOK^.

### TCR responsiveness of the Sil^ThPOK^ may be encoded by an evolutionarily conserved TF consensus site signature

To identify TFs that could be responsible for TCR responsiveness of the murine Sil^ThPOK^ during thymic development, we first assessed interspecies conservation of the *ThPOK* and *Cd4* silencers. This revealed that the Sil^ThPOK^ but not the Sil^CD4^ showed extensive sequence homology between all mammalian species examined^22^ (Supplementary Fig. 7a). Further, organization of TF consensus sites (as predicted by JASPAR algorithm) was well conserved between species for the Sil^ThPOK^, but not the Sil^CD4^ element. Thus 524 of 1315 (40%) predicted TF sites for the mouse Sil^ThPOK^ element were also conserved in relative position/orientation in placental and marsupial mammals (human versus opossum), compared to only 106 of 1071 (10%) for the mouse Sil^CD4^ element (Supplementary Fig. 7b)^22^. Given that precise motif grammar may not be critical for function of some cis elements (e.g., billboard enhancers), we compared just the relative number of sites for each TF within each element, regardless of position. We excluded TFs not expressed at the DP > SP transition (according to IMMGEN Skyline RNA-seq database), and not evolutionarily conserved (i.e., not predicted to bind both human and mouse homologs of either Sil^ThPOK^ or Sil^CD4^). For the remaining 170 TFs, the number of binding sites within Sil^ThPOK^ and Sil^CD4^ elements for three mammalian species (human, mouse, opossum) was averaged across all three species, and the ratio between Sil^ThPOK^ and Sil^CD4^ calculated for each TF, to reveal candidate TFs that may selectively bind Sil^ThPOK^ or Sil^CD4^. Importantly, TF sites that are overrepresented in the Sil^ThPOK^ versus the Sil^CD4^ include many TFs implicated in TCR signaling, such as Ebox factors (HEB, E2A)^23^, Egr1, NFAT, and NfkB (Supplementary Fig. 8a). Conversely, the Sil^CD4^ is enriched for different TF consensus sites that do not include sites for TFs implicated in TCR signaling (Supplementary Fig. 8b). The most enriched sites were mapped onto the respective sequence coordinates of the Sil^ThPOK^ or Sil^CD4^ to define distinct, evolutionarily conserved TF site signatures. Strikingly, the distribution of preferentially represented TF sites is closely conserved across species for the Sil^ThPOK^, but not for the Sil^CD4^ (Supplementary Fig. 8c). Of note, this does not imply that the latter are functionally unimportant, but rather that motif grammar (relative order and spacing of TF sites) may be more important for the function of the Sil^ThPOK^, than the Sil^CD4^.

### Sil^ThPOK^ regions responsible for lineage-specific silencing bind multiple effectors of TCR signaling

**Fig. 5:**
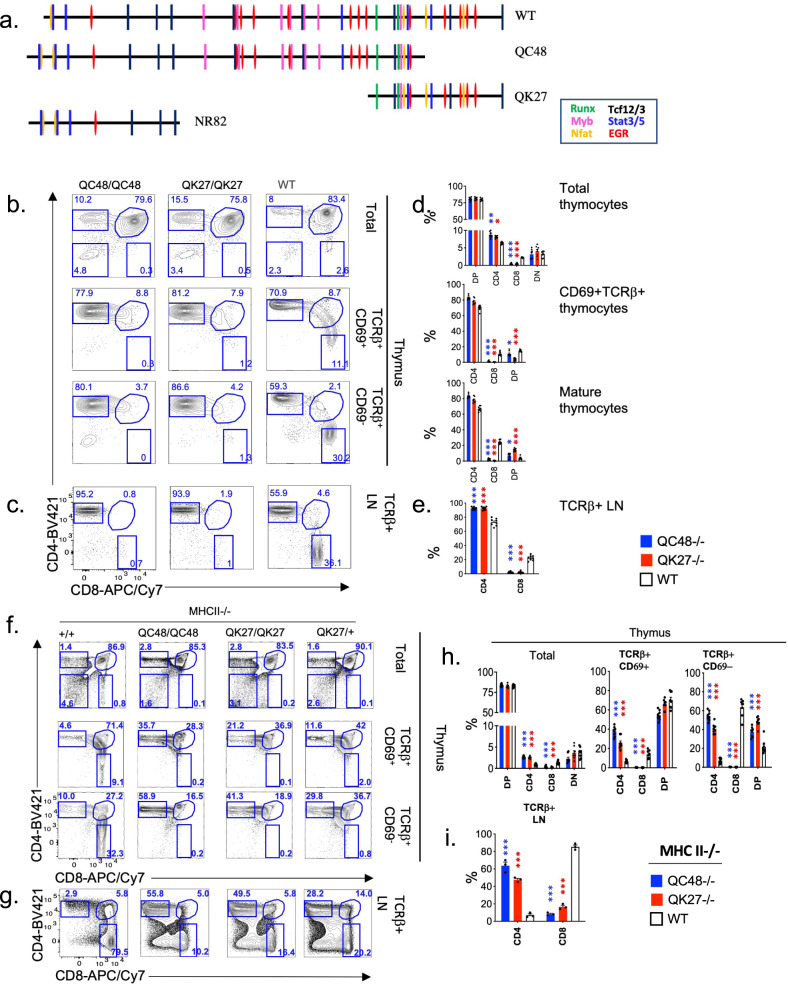
Distinct nonoverlapping regions of Sil^ThPOK^ are required for its silencing function. **a** Schematic of positions of transcription factor (TF) consensus binding sites within murine Sil^ThOPK^ (top row). Different TF motifs are color-coded according to the legend at the left. **b** FACS analysis of CD4, CD8a, TCRβ, and CD69 expression of total thymocytes (top and second rows), or CD4 and CD8a expression of indicated gated thymocyte subsets (bottom 2 rows) of ThPOK^sil.ΔQC48^, ThPOK^sil.ΔQK27^ and wt mice, as indicated. Note that mature CD8 population is absent in both mutant lines. **c** FACS analysis of TCR expression of total mesenteric lymph node (LN) cells (top row), or gated TCRβ + LN cells subsets (bottom row) of same strains of mice as above. Results are representative of multiple experiments. *N* = 3 independent animals per strain. **d** Plots showing % of DP, SP CD4, SP CD8, and DN thymocytes, or (**e**) SP CD4 and SP CD8 T cells from LN for mice of indicated genotypes. *N* = 5 independent animals per strain. Data are presented as mean values +/− SEM. A *P* value <0.05 was considered significant. Significant differences were determined by one-way ANOVA with post hoc Tukey HSD, and indicated by asterisks (*P < 0.01; **P < 0.005; ***P < 0.001). **f** FACS analysis of TCRβ, CD69, CD4, and CD8α expression by indicated thymic and (**g**) peripheral lymphocyte populations of ThPOK^sil.ΔQC48^, ThPOK^sil.ΔQK27^ and wt mice, as indicated, crossed to the MHC II-deficient background. **h**, **i** Plots showing % of DP, SP CD4, SP CD8, and DN thymocytes (**h**) or SP CD4 and SP CD8 T cells (**i**) for mice of indicated genotypes. *N* = 6 independent animals per strain. Data are presented as mean values +/− SEM. A *P* value < 0.05 was considered significant. Significant differences were determined between indicated mutant mice and WT control mice by one-way ANOVA with post hoc Tukey HSD, and indicated by asterisks (*P < 0.01; **P < 0.005; ***P < 0.001). Statistical significance was calculated for each indicated mutant line relative to wt mice (**d**, **e**), or MHC II−/− mice (**h**, **i**).

Before determining the role of specific TF binding to the Sil^ThPOK^ for its lineage-specific function, we first determined the essential region of the Sil^ThPOK^ required for its lineage and stage-specific function by creating three different Sil^ThPOK^ deletion mutant alleles: (A) Δ1–150 bp (line NR82); (B) Δ1–261 bp (line QK27); and (C) Δ357–418 bp (line QC48) (Fig. 5a). All three mutant mouse lines were bred to homozygosity. Strikingly, both QC48 and QK27 deletions resulted in a severe reduction in SP CD8 cells in both the thymus and periphery, suggesting the failure of silencing activity during thymic development (Fig. 5b–e). In contrast, homozygous mutant NR82 mice developed normal proportions of CD8 T cells in the thymus and periphery^24^. These results indicate that the regions deleted in lines QC48 and QK27, but not NR82, encode non-redundant functions essential for silencing. Next, we tested whether the absence of CD8 SP thymocytes in QC48−/− and QK27−/− mice reflected a block in the development of MHC class I-restricted thymocytes or redirection to the CD4 lineage by crossing each mutant onto a MHC class II-deficient background, which restricts development to class I-restricted cells. Strikingly, even on the MHC class II-deficient background QC48 and QK27 mice still generate CD4 T cells, in contrast to MHC II−/− control mice which develop only CD8 cells, suggesting redirection of class I-restricted T cells to the CD4 lineage (Fig. 5f–i). Collectively, nonoverlapping regions of the Sil^ThPOK^, i.e., 150–260 bp and 358–418 bp, are individually essential for lineage-specific Sil^ThPOK^ function. Notably, both of these regions are enriched for EGR consensus motifs, while the 358–418 bp region also includes two NFAT motifs.

**Fig. 6:**
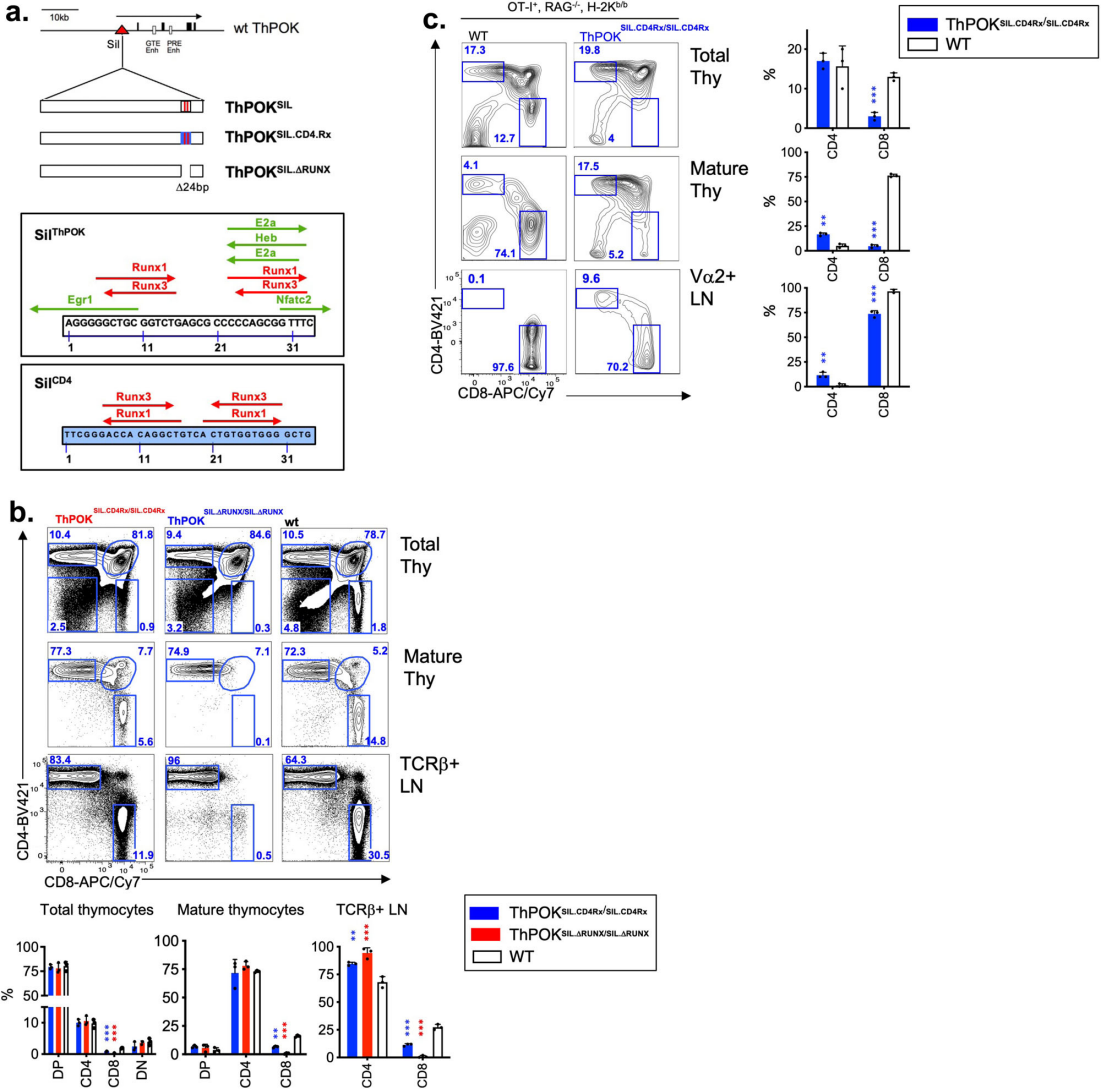
Region of Sil^ThPOK^ surrounding conserved Runx consensus sites is critical for its proper regulation. **a** Schematic of ThPOK^SIL.CD4.Rx^ and ThPOK^SIL.ΔRUNX^ knock-in alleles, indicating the location of conserved Runx sites (red bars). Blue color indicates swapped region from the Sil^CD4^. Bottom panels show the position/orientation of indicated TF consensus sites for the swapped regions. Note that the occurrence of Ebox consensus motifs at indicated positions within the Sil^ThPOK^ but not Sil^CD4^. **b** FACS analysis of CD4, and CD8 expression of total thymocytes, or indicated gated thymocyte and peripheral T-cell subsets of wt, CD4 ^SIL.CD4Rx/SIL.CD4Rx^ and CD4^SIL.ΔRUNX/SIL.ΔRUNX^ mice. Results are representative of multiple experiments. *N* = 3 independent animals per strain. Plots showing % of DP, SP CD4, SP CD8, and DN thymocytes for mice of indicated genotypes are shown at right. *N* = 3 independent animals per strain. Data are presented as mean values +/− SEM. A *P* value < 0.05 was considered significant. **c** FACS analysis of CD4 and CD8 expression by indicated thymic or peripheral lymphocyte populations of wt or CD4 ^SIL.CD4Rx/SIL.CD4Rx^ mice expressing the MHC class I-restricted OT-1 TCR transgene on the selecting H-2b/b background. Results are representative of multiple experiments. Note that the proportion of SP CD4 mature thymocytes is strongly or moderately increased for mature thymocytes a or gated peripheral T cells, respectively, from CD4 ^SIL.CD4Rx/SIL.CD4Rx^ mice. *N* = 3 independent animals per strain. Statistical significance was determined between indicated mutant mice and ThPOK+/+ mice by one-way ANOVA with post hoc Tukey HSD, and indicated by asterisks (*P < 0.01; **P < 0.005; ***P < 0.001). Statistical significance was calculated for each indicated mutant line relative to wt mice.

Of note, a 34-bp region containing conserved Runx-binding motifs, which have previously been shown to be essential for Sil^ThPOK^ function^8,9,25^, was left intact in both QC48 and QK27 mutants, so that their phenotypes are not attributable to disruption of Runx binding. Nevertheless, we noticed that this 34-bp region also encodes several predicted EGR, Ebox, and NFAT-binding sites (Fig. 6a). In order to evaluate the importance of these motifs, we generated further knock-in mice in which this 34-bp segment of the murine Sil^ThPOK^ was replaced with a 34-bp segment of the murine Sil^CD4^ that also contains two closely spaced Runx sites, but lacks any of the predicted Egr, Ebox or NFAT motifs (ThPOK^SIL.CD4Rx^ mice) (Fig. 6a). Importantly, ThPOK^SIL.CD4Rx^ mice showed a striking reduction in SP CD8 thymocytes and CD8 peripheral T cells, albeit less severe than control ThPOK^SIL.ΔRUNX^ mice in which both Runx sites are deleted (Fig. 6b). Crossing ThPOK^SIL.CD4Rx^ mice to OT-1 TCR transgenic mice reveal substantial redirection of class I-restricted thymocytes to the CD4 lineage, indicating aberrant ThPOK upmodulation as a result of impaired silencer function (Fig. 6c). Hence, the presence of Runx motifs at this location within the Sil^ThPOK^ is not sufficient to confer normal regulation of silencer activity. Rather other motifs surrounding the Runx sites, including predicted Egr, Ebox, and NFAT sites, are also critical, suggesting functional synergy between these factors and Runx factors in the control of Sil^ThPOK^ activity.

**Fig. 7:**
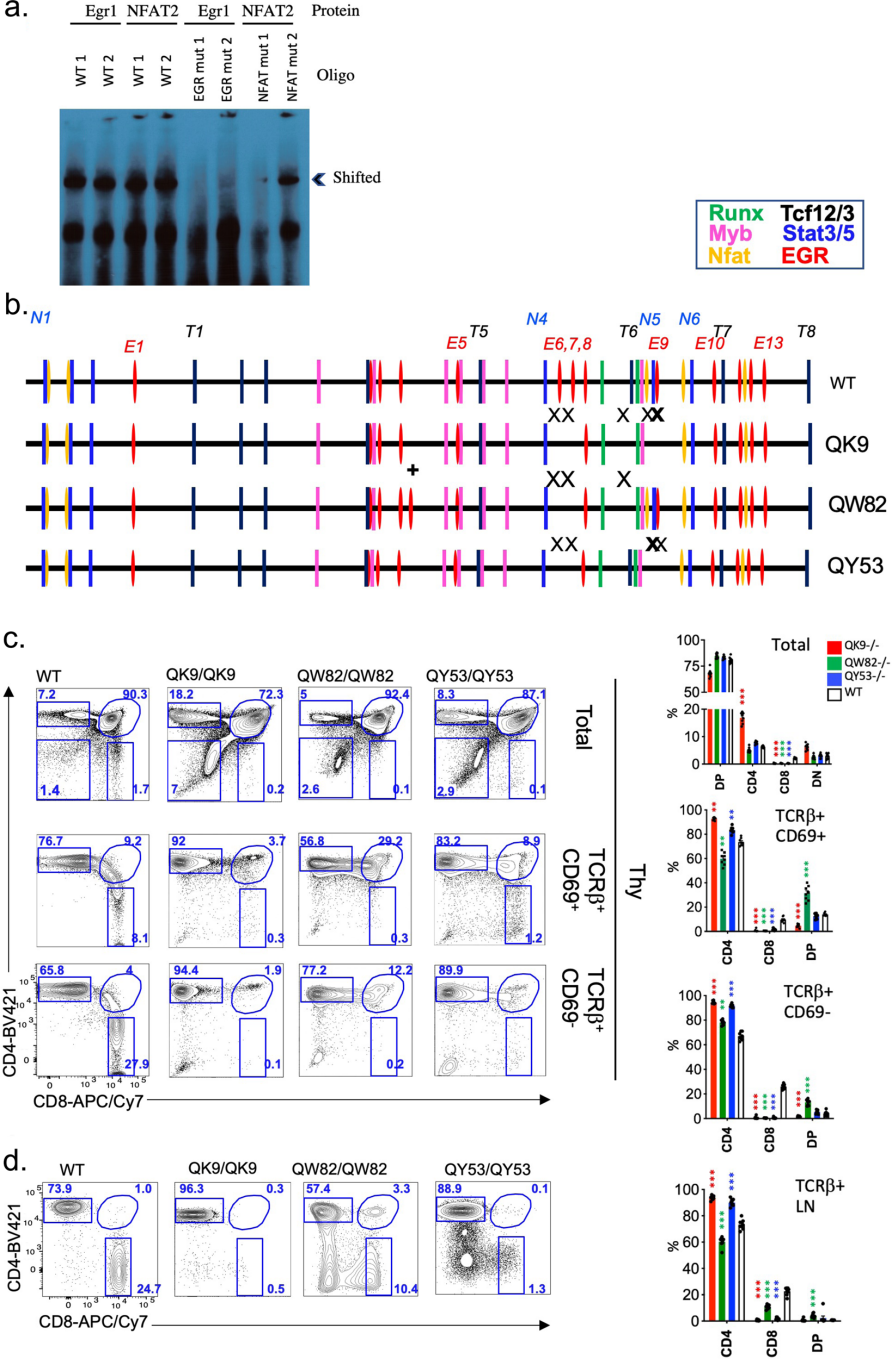
Mutation of EGR and NFAT-binding motifs adjacent to conserved Runx sites impairs Sil^ThPOK^ function. **a** EMSA analysis showing EGR1 and NFAT2 binding to oligos comprising wt silencer sequence (region surrounding Runx sites), or mutated at consensus EGR or NFAT motifs, as indicated. **b** Position of TF consensus binding sites for wt silencer, or indicated variant alleles. FACS analysis of TCRβ, CD69, CD4, and CD8 expression of total thymocytes, gated thymocytes (**c**), and peripheral T-cell subsets (**d**) of wt mice or mutant lines, as indicated. Results are representative of multiple experiments. *N* = 6 independent animals per strain. Data are presented as mean values +/− SEM. A *P* value < 0.05 was considered significant. Statistical significance was determined between indicated mutant mice and ThPOK+/+ mice by one-way ANOVA with post hoc Tukey HSD, and indicated by asterisks (*P < 0.01; **P < 0.005; ***P < 0.001). Statistical significance was calculated for each indicated mutant line relative to wt mice.

Given that regions required for Sil^ThPOK^ function are notably enriched for EGR and NFAT consensus motifs, we directly tested whether EGR and NFAT can bind to the Sil^ThPOK^ by EMSA. We used two different labeled oligos corresponding to 161–268 bp (probe 1) and 169–371 bp (probe 2) regions of the Sil^ThPOK^. For each region, we generated alternate probes encoding either the endogenous Sil^ThPOK^ sequence (wt probes), or mutant sequences in which consensus Egr or NFAT motifs (as determined by JASPAR algorithm) are disrupted (mutant probes). We observed that (1) both wt probes bind to EGR1 and NFAT2 factors, as evidenced by band shift upon incubation with cell lysates overexpressing either factor, while (2) mutant probes failed to undergo such band shift, or to a much lesser extent, demonstrating site-specificity of this TF binding (Fig. 7a).

### Effect of combinatorial disruption of EGR, NFAT, and Ebox motifs on Sil^ThPOK^ function

Next, to test the in vivo relevance of EGR, Ebox (TCF12/3), and NFAT motifs for Sil^ThPOK^ function, we generated a series of mutant mouse lines in which we mutated different combinations of these sites (numbered as shown in Fig. 7b) within the context of the endogenous full-length Sil^ThPOK^ element: (1) the QK9 allele, containing mutations of three EGR (E6, E7, E9), one Ebox (T6), and one NFAT (N5) site, (2) the QW82 allele, containing mutations of two EGR (E6, E7), and one Ebox (T6) site, and (3) The QY53 allele, containing mutations of three EGR (E6, E7, E9), and one NFAT (N5) (Fig. 7b).

**Fig. 8:**
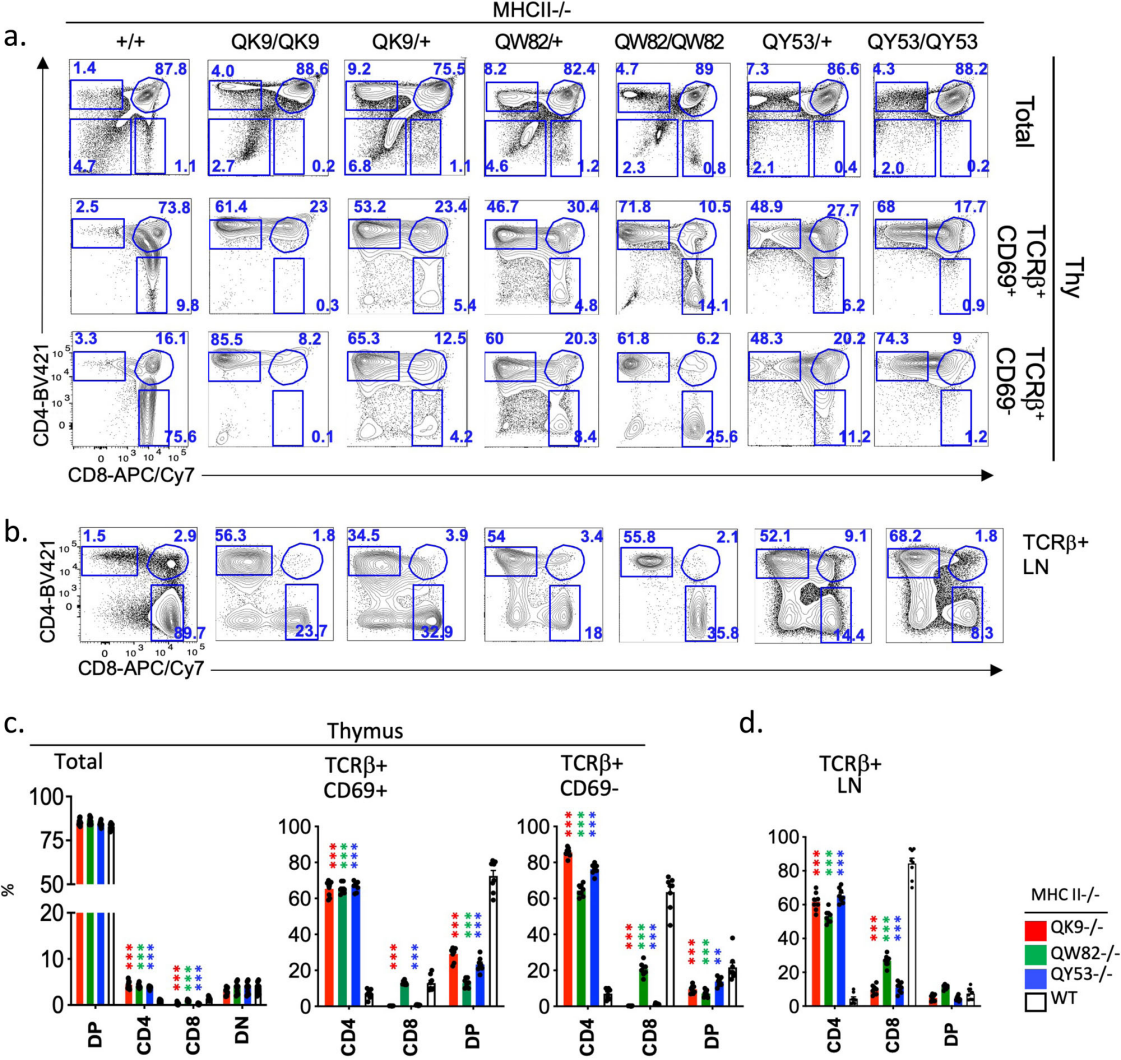
Mutation of EGR and NFAT-binding motifs adjacent to conserved Runx sites impairs normal lineage choice of MHC class I-restricted thymocytes. **a**, **b** FACS analysis of TCRβ, CD69, CD4, and CD8 expression of total thymocytes, gated thymocytes (**a**), and peripheral T-cell subsets (**b**) of wt, or mutant mouse lines crossed to MHC II−/− background. **c**, **d** Plots showing % of DP, SP CD4, SP CD8, and DN thymocytes for mice of indicated genotypes. *N* = 6 independent animals per strain. Data are presented as mean values +/− SEM. A *P* value < 0.05 was considered significant. Statistical significance was determined between mutant mice and ThPOK+/+ mice on MHC II−/− background by one-way ANOVA with post hoc Tukey HSD, and indicated by asterisks (*P < 0.01; **P < 0.005; ***P < 0.001). Statistical significance was calculated for each indicated mutant line relative to wt mice.

All mutants showed major defects in thymic development: (1) Homozygous QK9−/− mice completely lack CD8 T cells in thymus and periphery, suggesting loss of Sil^ThPOK^ function (Fig. 7c, d). Furthermore, crossing to MHC class II-deficient background to limit thymocyte development to class I-restricted cells, showed the preferential generation of CD4 rather than CD8 T cells, compared to MHC II−/− control mice, indicating redirection of class I-restricted thymocytes to the CD4 lineage, presumably consequent to aberrant ThPOK upmodulation (Fig. 8a, b). (2) Homozygous QW82 mice display a similar phenotype to QK9 mice, i.e., lack of CD8 T-cell development, and substantial redirection of class I-restricted cells to the CD4 lineage, as evidenced by the presence of a large proportion of CD4 T cells in class II- deficient QW82−/− mice (Fig. 8a–d), suggesting a severe defect of Sil^ThPOK^ repression. (3) Homozygous QY53−/− mouse also displays a striking block in CD8 development and redirection of a large proportion of class I-restricted thymocytes to the CD4 lineage as QK9 and QW82 mice (Figs. 7c, d and 8a–d). Collectively, our mutational analysis indicates that (a) two EGR consensus motifs that are mutated in all three mutant lines (E6, E7 sites; Fig. 7a) are indispensable for CD8 lineage-specific ThPOK silencing, while (b) NFAT, Ebox, and EGR sites (N3, E9, and T6 sites) that are mutated in only some lines are unnecessary or redundant for this process. Of note, while the QC48 deletion showed a severe developmental phenotype (Fig. 5), this may not be attributable to Egr or Nfat sites contained within this region, as mutating all Egr sites (line TO61) or all Nfat sites (line RQ17) within this region had no effect on CD4/CD8 ratio. It remains possible that Egr and NFAT sites within the QC48 region are collectively required for correct lineage choice.

### Effect of disruption of specific NFAT and Ebox motifs on Sil^ThPOK^ function

**Fig. 9:**
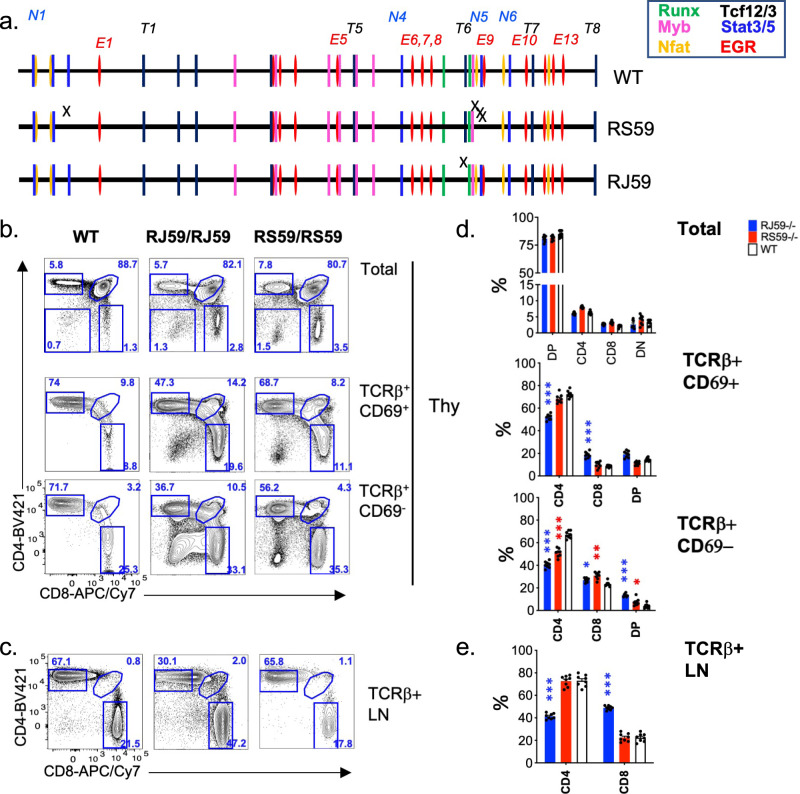
Mutation of HEB/E2A binding motifs adjacent to conserved Runx sites enhances Sil^ThPOK^ function. **a** Position of TF consensus binding sites for wt silencer, or indicated mutant RJ59 and RS59 alleles. FACS analysis of TCRβ, CD69, CD4, and CD8 expression of (**b**) total thymocytes, gated thymocytes and (**c**) peripheral T-cell subsets of wt mice or mutant lines, as indicated. **d**, **e** Plots showing % of DP, SP CD4, SP CD8, and DN thymocytes for mice of indicated genotypes. *N* = 6 independent animals per strain. Data are presented as mean values +/− SEM. A *P* value < 0.05 was considered significant. Statistical significance was determined between mice of ThPOK ^SIL.CD4Rx/SIL.CD4Rx^ and ThPOK+/+ genotypes by one-way ANOVA with post hoc Tukey HSD, and indicated by asterisks (*P < 0.01; **P < 0.005; ***P < 0.001). Statistical significance was calculated for each indicated mutant line relative to wt mice.

To separately evaluate the contribution of these NFAT and Ebox factor binding sites to silencer activity independent of EGR site mutations, we generated two additional mutant alleles, with mutations of (A) Ebox site T6 (line RJ59;), or (B) NFAT site N3, which also affects adjoining Stat and EGR motifs (line RS59) (Fig. 9a). As expected from our previous mutants, neither homozygous line induced skewing toward the CD4 lineage. Surprisingly, however, they displayed substantial skewing toward the CD8 lineage in the thymus, resulting in CD4:CD8 ratios of 1.3:1 and 1.8:1 for homozygous RJ59−/− and RS59−/− mice, respectively (versus 3:1 in wt mice) (Fig. 9b–d). In addition, there was a notable increase in atypical mature DN T cells in the thymus (Fig. 9b). Similar CD8 skewing was evident in peripheral T cells of Ebox mutant RJ59−/− mice, but not NFAT mutant RS59−/− mice (Fig. 9c, e). We did not observe substantial misdirection of either class I- or class II-restricted cells in RJ59−/− or RS59−/− mice crossed to MHC II and MHC I-deficient backgrounds, suggesting that reduction in ThPOK expression in these lines may occur too late in development to allow CD8 commitment. Altogether, these data suggest that Ebox and NFAT binding to specific sites near conserved Runx-binding sites of the Sil^ThPOK^ is necessary to oppose silencer function during the development of class II-restricted thymocytes to the CD4 lineage.

### CD4 commitment involves intra-locus chromatin looping between the Sil^ThPOK^ and other cis-regulatory elements

**Fig. 10:**
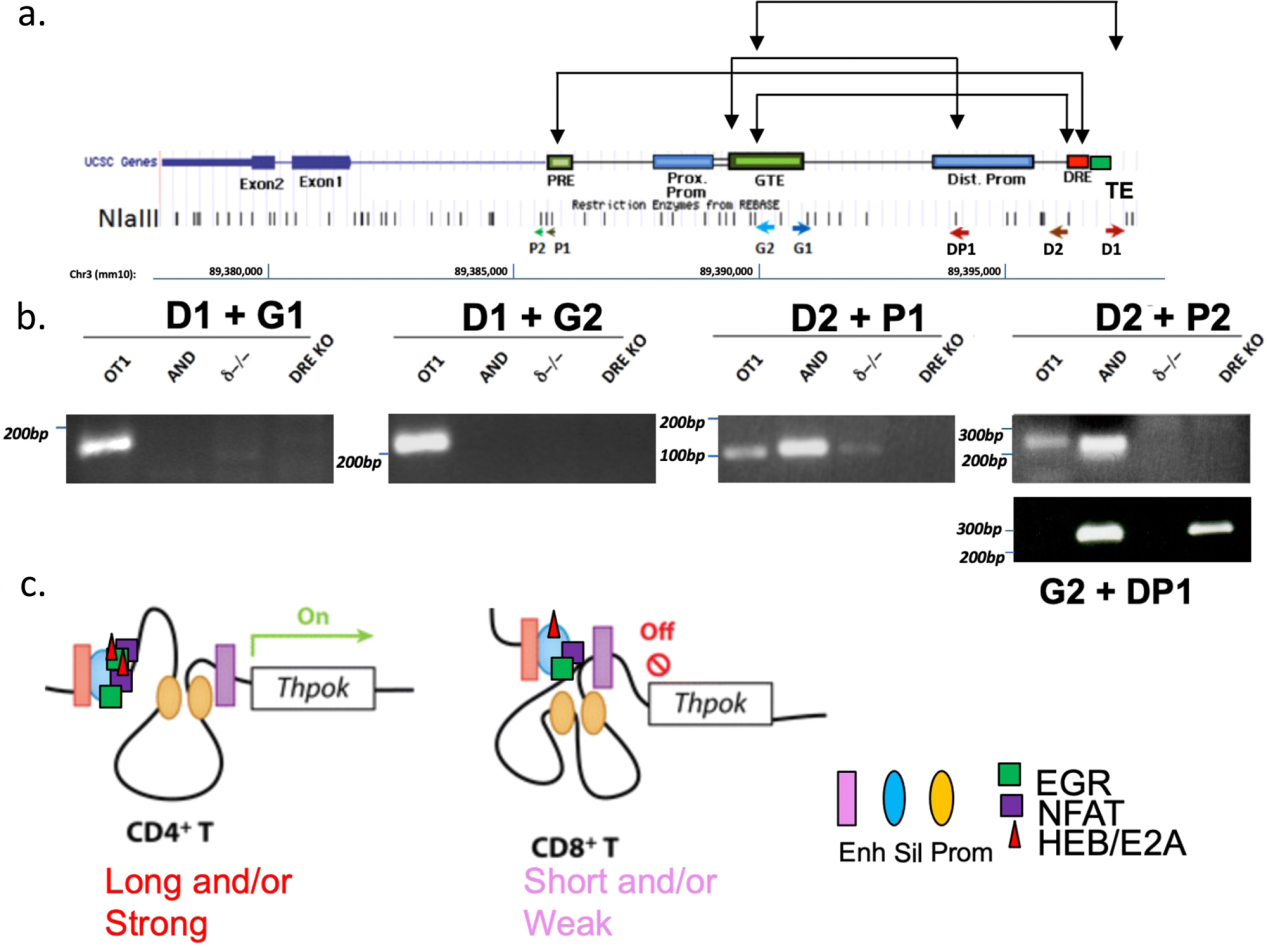
Chromosome conformation capture (3C) reveals altered proximity of Sil^ThPOK^ with other *ThPOK* cis elements in MHC class I- versus class II-restricted thymocytes. **a** Schematic of *ThPOK* gene organization showing exons (dark blue boxes), promoters (light blue boxes), enhancers (green boxes), and silencer (red box). Arrowheads at bottom of the panel indicate the position/orientation of PCR primers, while connected arrows at top of the panel show different primer combinations that were utilized. **b** 3C analysis of physical interactions of Sil^ThPOK^ with other *ThPOK* cis elements in total thymocytes from AND Tg+ (class II-restricted), OT-1 Tg+ (class I-restricted), CD3δ−/− (uncommitted; blocked at DP stage), and ThPOK^ΔSil^ (DRE knockout) mice, respectively. *N* = 3 independent animals per strain. **c** Model of proposed *ThPOK* cis element interactions in class I- versus class II-restricted thymocytes.

Given that enhancers and silencers function in part by controlling chromatin topology^26,27^, we asked whether topological assembly of *ThPOK* regulatory regions may be regulated in a stage- and/or lineage-specific manner. Accordingly, we performed 3C analysis on different thymocyte populations, i.e., predominantly class I-restricted thymocytes from OT-1 transgenic mice, predominantly class II-restricted thymocytes from AND transgenic mice, unsignaled DP thymocytes from CD3δ−/− thymocytes, and ThPOK^Δsil^ thymocytes which express ThPOK constitutively due to absence of the Sil^ThPOK^ (Fig. 10a, b). Interestingly, we observed a selective interaction between Sil^ThPOK^ with the lymphoid enhancer (GTE) in OT-1 transgenic thymocytes, correlating with transcriptional repression of *ThPOK*. Conversely, interaction between the lymphoid enhancer (GTE) and the distal promoter was only observed in AND transgenic and ThPOK^Δsil^ thymocytes, correlating with transcriptional activation of *ThPOK*. These observations suggest that the Sil^ThPOK^ may specifically sequester the GTE enhancer away from the distal promoter in class I-restricted thymocytes (Fig. 10c). Analysis of publicly available databases indicates striking stage- and lineage-specific changes in chromatin accessibility and histone modification of the Sil^ThPOK^, GTE enhancer, and distal promoter during thymic development (Supplementary Fig. 9a, b).

## Discussion

CD4 versus CD8 lineage commitment is controlled by TCR specificity, such that long and/or strong TCR signals elicited by MHC class II lead to CD4 commitment, whereas weak and/or transient signals elicited by MHC class I lead to CD8 commitment. But how differential TCR signals culminate in alternate transcriptional programs driving CD4 versus CD8 choice has remained an open question. In this study, using a regulatory element swap approach, we provide compelling evidence that the Sil^ThPOK^ encodes the intrinsic capacity to respond to TCR signaling leading to MHC restricted lineage choice, and show how this occurs at the molecular level.

Our comparison of the Sil^ThPOK^ and Sil^CD4^ elements shows that while they share the ability to suppress gene transcription in committed CD8 cells, they function very differently in early thymocyte developmental stages, particularly at the DP and CD4 + 8^lo^ stages. Thus, these silencers possess intrinsically distinct functional capacities regardless of the genomic context, and dominantly control other cis elements in both endogenous and exogenous gene contexts. For example, the Sil^ThPOK^ suppresses the transcription of *Cd4* in DP thymocytes of CD4^ThPOK.Sil^ mice similar to the suppression of endogenous *ThPOK* in DP thymocytes of wt mice. Conversely, derepression of *ThPOK* transcription in DP thymocytes of ThPOK^CD4.Sil^ mice mirrors expression of endogenous *Cd4* in DP thymocytes of wt mice. Hence, differential stage-specific regulatory control of the *ThPOK* and *Cd4* loci appears to be autonomous and encoded primarily within their respective silencers.

The shared ability of Sil^ThPOK^ and Sil^CD4^ elements to repress transcription following CD8 lineage commitment may largely reflect the presence of evolutionarily conserved Runx-binding motifs in both silencers, which have previously been shown to be critical for the activity of both the Sil^CD4^ and Sil^ThPOK^ elements^9,14,16,28,29^. Nevertheless, the mechanism by which Runx binding supports silencing is not well understood. ChIP analysis shows binding of Runx complexes (Runx1 or 3) to the Sil^ThPOK^ at all stages of thymic development, regardless of whether ThPOK is expressed, suggesting that the TCR responsiveness of the ThPOK silencer requires Runx factors to synergize with other factors bound to the Sil^ThPOK^. Our finding that knocking the Runx motifs from the Sil^CD4^ into the Sil^ThPOK^ (ThPOK^SIL.CD4.Rx^ mice) causes aberrant redirection of class I-restricted thymocytes to the CD4 lineage, indicates that precise TF-binding site syntax surrounding the Runx sites is critical for Sil^ThPOK^ function, and that the mere presence of Runx sites even at the same relative position of the silencer is not sufficient. Interestingly, some thymocytes still develop to the CD8 lineage in ThPOK^SIL.CD4.Rx^ mice, compared to mice which lack Runx sites, indicating that silencing still occurs for some cells, possibly those expressing particularly low-affinity TCRs. We conclude that the regions surrounding the Runx sites of the Sil^ThPOK^ and Sil^CD4^ elements recruit different TFs that critically control stage-specific activity of these elements. Our analysis reveals that motifs for TFs implicated in TCR signaling are preferentially represented in the Sil^ThPOK^ versus Sil^CD4^, and that disrupting these motifs perturbs normal regulation of silencing, leading to abnormal CD4-8 development. In particular, specific EGR sites nearby to conserved Runx-binding sites are necessary to promote silencer function and oppose CD4 development, while certain Ebox and NFAT sites are necessary to oppose silencer function and permit CD4 development.

There is substantial data to support an important role of TCR signaling in the induction of EGR^30^, NFAT^31^, and Ebox factors^32^. EGR factors have in turn been implicated in CD4 development^33–35^ and regulation of Th differentiation and function^36^. NFATc proteins are induced strongly in CD4 compared to DP and CD8 thymocytes^37^, and have been previously implicated in the control of positive selection^38,39^. Finally, we previously showed that conditional deletion of HEB/E2A at the DP stage leads to a severe defect in CD4 T-cell development, while deletion of the E protein antagonists Id2 and Id3 at the DP stage favors CD4 over CD8 lineage development^40^. Our current data provide a partial molecular genetic mechanism for some of these effects. Targeting specific Ebox and NFAT sites in silencer (T6/ N5) causes loss of mature CD4 thymocytes, and gain of mature DN and CD8 thymocytes, indicating increased silencing activity, which results in diminished ThPOK expression and consequent loss of CD4 expression. So HEB and NFAT factors appear to play a role in tuning Sil^ThPOK^ activity in response to different TCR signals, such that strong/long TCR signals elicited in MHC class II-restricted thymocytes cause preferential association of HEB and NFAT factors to the Sil^ThPOK^ leading to its loss of activity. Cooperative interaction of Ebox and NFAT factors has been established in other cellular contexts^41^. On the other hand, targeting specific EGR sites in the silencer (E6 / E7) drives the development of all thymocytes to the CD4 lineage, indicating blockade of silencer function and constitutive ThPOK expression. Hence, it appears that even weak class I-restricted signals are sufficient to trigger EGR binding to these sites and EGR binding to Sil^ThPOK^ is obligatory for silencing function. The effect of mutation of these EGR sites appears to be dominant over the effect of mutation of Ebox and NFAT-binding sites (since QK9, QW82, and QY53 mice have both E6/E7 sites and one or more T6/N5 sites mutated). It’s been shown that CD4-inducing TCR signals result in increased Egr1 and Egr2 induction versus CD8-inducing signals^42^. So, EGR factors induced by TCR signals are necessary to actively turn on silencer activity but may not be involved in tuning silencer activity in response to differential TCR signaling.

Analysis of available ATAC-seq data from purified thymocyte subsets indicates that the Sil^ThPOK^ region is accessible in DN1-DP stages, suggesting that it is poised for activity, but that this accessibility is substantially lost in both CD4 and CD8 thymocytes (Supplementary Fig. 9a). We suggest that this loss of accessibility is due to dense binding by TFs that are induced during positive selection and lineage commitment, including NFAT, Ebox, and EGR factors. Based on ATAC data, it further appears that the *ThPOK* distal promoter and GTE enhancer become markedly more accessible during the transition from the DP to SP CD4 stage, and that the GTE simultaneously comes into close proximity with the distal *ThPOK* promoter according to 3C analysis. These changes do not occur in class I-restricted cells, where the GTE instead loops to the Sil^ThPOK^. Sil^ThPOK^-deficient cells which instead exhibit constitutive interaction of distal promoter and lymphoid enhancer (GTE), suggesting that weak TCR signals promote differential silencer-dependent chromatin organization at the *ThPOK* locus. In the context of our mutational analysis, one might hypothesize that EGR factors promote looping of the silencer to the GTE, while Ebox/NFAT factors may oppose it.

CD4 versus CD8 lineage commitment is controlled by TCR signals of different strength and duration, which in turn promote different downstream patterns of TF expression. It appears that initially many/most of the TFs involved in CD4 vs CD8 commitment are induced by all TCR signals, e.g., Egr, NFAT, etc., but that their kinetics of expression may differ in response to TCR signals of different strength/duration. As a consequence, there will be differences in the level and/or duration of binding of these shared TFs to the *ThPOK* silencer. By mutating particular consensus motifs within the silencer, we have prevented the binding of cognate TFs to these sites, and potentially affected the recruitment of other TFs to nearby sites. The fact that some silencer mutations result in intermediate phenotypes is consistent with a multilayered and redundant control mechanism. For instance, in QW82 mutants some class I-restricted thymocytes differentiate to the CD8 lineage while others progress aberrantly to the CD4 lineage. Given that thymocytes are heterogeneous in terms of their surface TCR affinity/avidity to the MHC I and MHC II encoded by their clonotypic TCRs, it’s reasonable to speculate that those class I-restricted thymocytes which are still able to differentiate to the CD8 lineage in QW82 mice express particularly low-affinity TCRs, resulting in distinct TF-binding patterns at the ThPOK silencer that are able to maintain its activity. Finally, it’s entirely possible that other non-TCR-dependent signal pathways also impinge on the ThPOK silencer and contribute to control of its activity, including the IL-7 signal pathway which has been implicated in CD8 commitment^43^. Indeed, we have identified consensus Stat factor binding sites in the silencer, and in the future will test how TCR signals may cooperate with or antagonize IL-7 signal to control silencer activity and lineage choice.

In summary, we postulate that stage-specific Sil^ThPOK^ activity is controlled by TFs that are transcribed/activated downstream of TCR signals, including EGR, NFAT, and Ebox factors, which in turn control topological organization of in the *ThPOK* regulatory region particularly the apposition of the Sil^ThPOK^ with other cis elements.

## Methods

### Mice

RAG1−/− (stock # 034159), β2m−/− (stock # 002087), and AND TCR transgenic (stock # 002761) mice have been procured from Jackson Laboratory. OT-1 TCR transgenic RAG2−/− (stock # 2334) and MHC II−/− (stock # ABBN12) mice were obtained from Taconic. CD3δ−/− mice are from our own colony^44^. All other mouse lines described in this paper have been generated by the FCCC Transgenic Facility on the C57BL/6J strain of *M. musculus*. Animals used in all experiments were 6–10 weeks of age, and males and females were used in equal proportions (no difference was noted between males and females in any experiment). Animal care was in accordance with NIH guidelines. Mice were maintained on a 12 h light/dark cycle, at 75 °F and 50% humidity. All experimentation involving animals was approved by the Institutional Animal Care and Use Committee (IACUC) of Fox Chase Cancer Center.

### In vivo treatment with anti-TCRβ mAb

CD4^ThPOKsil/ThPOKsil^ were crossed to MHC II −/− mice to generate compound mutant CD4^ThPOKsil/ThPOKsil^ MHC II −/− mice. Four to six-week-old animals were injected i.p. with 30 μg of azide-free anti-TCRβ mAb H57-597 and thymocytes isolated 15 h later.

### ZFN-mediated gene targeting in mouse embryos

Site-specific mutagenesis was carried out, according to established procedures^22^. Briefly, a pair of ZFN RNAs that recognize a specific target site near the Sil^ThPOK^ was designed and generated by Millipore-Sigma (Genome Editing division). The ZFN target sequence is ACCGCTACCCTAACCcataaCTGGAAGGGGTTTAG (capital letters denotes nucleotides actually bound by right and left ZFN proteins). mRNAs encoding the two site-specific ZFNs (50 ng/μl) were introduced together with double-stranded DNA-targeting constructs bearing the desired mutations/deletions into 1-cell C57BL/6J mouse oocytes by pronuclear injection, and injected oocytes were transferred to a pseudopregnant surrogate mother. Targeting constructs contained 1.5 and 0.8 kb arms of homology on either side of the desired mutations/deletion. Positive founder pups were identified based on the reduced size of PCR product using primers F1 (5′-ATCCCTACGAAGAAGCCTCT-3′) and R1 (5′-AGGCTTTCCATGTCAGGGTC-3′), and mated to C57BL/6 mice for seven generations to produce stable heritable knock-in lines.

### Flow cytometry

All fluorescently labeled antibodies used were obtained from commercial sources (eBioscience, Biolegend, BD, or Invitrogen), including TCRβ-PE/Cy5 (H57-597 Cat # 109210; Lot # B170070), CD4-BV421 (clone RM4-5; BioLeg Cat # 100544; Lot # B293278), CD8a-APC/Cy7 (clone 53-6.7; BioLeg Cat # 100714; Lot # B276265), CD69-PE/Cy7 (clone H1.2F3; eBio Cat # 25-0691-82; Lot # E07583-1635), CD24-FITC (clone M1-69; BioLeg Cat 101806; Lot # B184710), CD62L-PerCP/Cy5.5 (clone MEL-14; BioLeg Cat 104432; Lot # B272105). 1 × 10^6^ cells were stained in 100 μl of PBS, 5% FCS at 4 °C for 30 min with 0.5 μg/ml of each antibody in 96-well round-bottomed microtiter plates, cells washed three times by centrifugation at 1500 rpm for 5 minutes, and then resuspended in 200 μl of PBS, 5% FCS. In total, 5 µL of PI solution (10 μg/mL PI in PBS) were added to each sample just prior to analysis. Dead cells, doublets, and debris were excluded in all analyses. Flow cytometry analyses were conducted on a FACS LSRII. Cell sorting was performed on a FACSAria II (Becton, Dickinson, and Company). FACS data were collected using FACS Diva version 7.0 or 9.0, and data were analyzed using FlowJo software (versions 9.3.3, 10.1, or 10.2, FlowJo, Ashland, OR, USA). In contour plots, expression levels were shown at 5% probability, unless indicated otherwise in the figure legend. Total thymus cell counts were performed for each animal in presence of trypan blue, showing that none of the silencer mutations alter the absolute number of total thymocytes relative to WT mice (1.6 × 10^8^ +/− 0.3 for 6–10-week-old mice). Furthermore, the frequency of pre-selection (TCRlo CD69−) DP thymocytes is unaffected in any mutant (except for CD4^ThPOK.Sil^ mice), excluding the possibility that mutations of *ThPOK* silencer cause reduction of these precursors. Immature signaled (TCRβ+ CD69+) and mature (TCRβ+ CD69−) thymocytes are defined according to the gating strategy shown in Supplementary Fig. 10.

### EMSA

Nuclear extracts were prepared from human embryonic kidney (HEK) 293T cells cultured on flat-bottom six-well cell culture plates were transfected with Flag-tagged murine Nfat2 or Egr1 constructs (cloned into the pcDNA3 expression vector) in the presence of 10 µg/ml polybrene. Negative controls included nuclear extracts from cells transfected with vector alone. TF expression was verified by immunoblot analysis and used as a protein source for binding assay. DNA-binding probes were generated by annealing of synthetic double-stranded oligonucleotides corresponding to the target region and end-labeling with polynucleotide kinase and digoxigenin-11-ddUTP using EMSA Kit (Sigma). The anti–Flag Ab (Sigma) was used for ‘supershifting’ of TF protein–DNA complexes.

### Quantitative RT-PCR

Cell sorting was carried out using a BD FACSAria and FACS Diva software. 100,000 cells were sorted directly into RNA lysis buffer (4 M guanidinium thiocyanate, 25 mM sodium citrate, pH 7.0, 0.5% (wt/vol) N- laurosylsarcosine (Sarkosyl) and 0.1 M 2-mercaptoethanol)^45^. cDNA was synthesized using the High Capacity RNA-to-cDNA kit (ThermoFisher). For mouse Cd4, we used commercial Taqman (probe-based) assay Mm00442754_m1 (Life Technologies), with the QuantStudio6 thermocycler (Life Technologies) and QuantStudio Realtime PCR Software.

For mThPOK, we performed quantitative RT-qPCR using SYBR Green Master Mix (ThermoFisher) and our own forward and reverse primers^8^:

ThPOK For 5′-ACCCAACGGCTGAAAGGA-3′

ThPOK Rev 5′-GCTGCTGTGGTCTGGCAAT-3′

Transcript levels are expressed as 2^(-J), where J refers to the difference between Ct (transcript of interest) and Ct (Rps6).

### 3C analysis

Chromatin crosslinking was performed by adding 9.5 ml of 2% formaldehyde/10% FCS/PBS per 1 × 10^7^ thymocytes from AND Tg+ (class II-restricted), OT-1 Tg+ (class I-restricted), CD3δ−/− (uncommitted; blocked at DP stage), and ThPOK^ΔSil^ (DRE knockout) mice, followed by incubation at room temperature for 10 min. The crosslinking reaction was quenched by the addition of 1.425 ml of 1 M glycine (ice cold). Cells were isolated by spinning for 8 min at 225 × *g* at 4 °C, and supernatants were carefully removed. Cell pellets were resuspended in 5 ml cold lysis buffer (10 mM Tris-HCl, pH 7.5; 10 mM NaCl; 5 mM MgCl_2_; 0.1 mM EGTA; 1 × complete protease inhibitor; 11836145001 Roche) and incubated for 10 min on ice, and nuclei isolated by centrifugation at 400 × *g* for 5 min. Quantitative analysis of chromosome conformation capture assays was performed^46^ using 4 BP cutter NlaIII and *ThPOK* BAC plasmid was used as the positive control.

### Statistics and reproducibility

No statistical method was used to determine the sample size. Instead, sample sizes were rationalized by weighing sufficient replication (to determine the extent of biological variation) with reduction of total animals used. Data were excluded only for technical reasons, such as low cell viability. Regarding replication, all in vivo analyses were performed on a total of three to six animals per genotype (across at least three separate experiments). All attempts at replication were successful. Randomization was not used; assignment to experimental groups was based on genotype. To exclude physiological and environmental covariates, mice of different genotypes were derived from the same litters as control mice (as much as possible), or cohoused prior to analysis. Statistical analysis for nonsequencing data was performed using GraphPad Prism software. Data were analyzed by applying one-way ANOVA with post hoc Tukey HSD (Honest Significant Difference) method. A *P* value of less than 0.05 was considered significant. *P < 0.05, **P < 0.01, ***P < 0.001.

### Reporting summary

Further information on research design is available in the Nature Research Reporting Summary linked to this article.

## Supplementary information

Supplementary Information

Description of Additional Supplementary Files

Supplementary Data 1

Reporting Summary

## Data Availability

The source data of graphs are provided as Supplementary Data 1. Uncropped and unedited blot/gel images corresponding to Figs. 7a and 10b are provided as Supplementary Figs. 11 and 12, respectively. All other data are available from the authors upon reasonable request.
