# Supplementary material for: An autonomous TCR signal-sensing switch influences CD4/CD8 lineage choice in mice
Source: Commun Biol. 2022 Jan 21;5:84. doi: 10.1038/s42003-022-02999-5 (PMC8783009; doi:10.1038/s42003-022-02999-5)
Supplement: Supplementary file 1 — Supplementary Information [file 42003_2022_2999_MOESM1_ESM.pdf]

SUPPLEMENTARY INFORMATION.

An autonomous TCR signal-sensing switch influences CD4/CD8 lineage choice in mice.

Jayati Basu[1], Jikun Zha[1], Emmanuelle Nicolas[1], Michael Coulton[1], Philip Czyzewicz[1], Xiang Hua[1], Lu Ge[1], Dietmar J. Kappes [1*]

[1]Fox Chase Cancer Center, 333 Cottman Avenue, Philadelphia, PA 19111, USA

Running title: TCR-sensing switch

* To whom correspondence should be addressed: Dietmar J. Kappes, Ph.D., Fox Chase Cancer Center, 333 Cottman Avenue, Philadelphia, PA, 19111, USA, Telephone 215 728 5374, e-mail, Dietmar.Kappes@fccc.edu

Keywords: ThPOK, CD4, TCR signaling, lineage choice, bistable switch, silencer

Supplementary Figures.

Suppl. Fig. 1

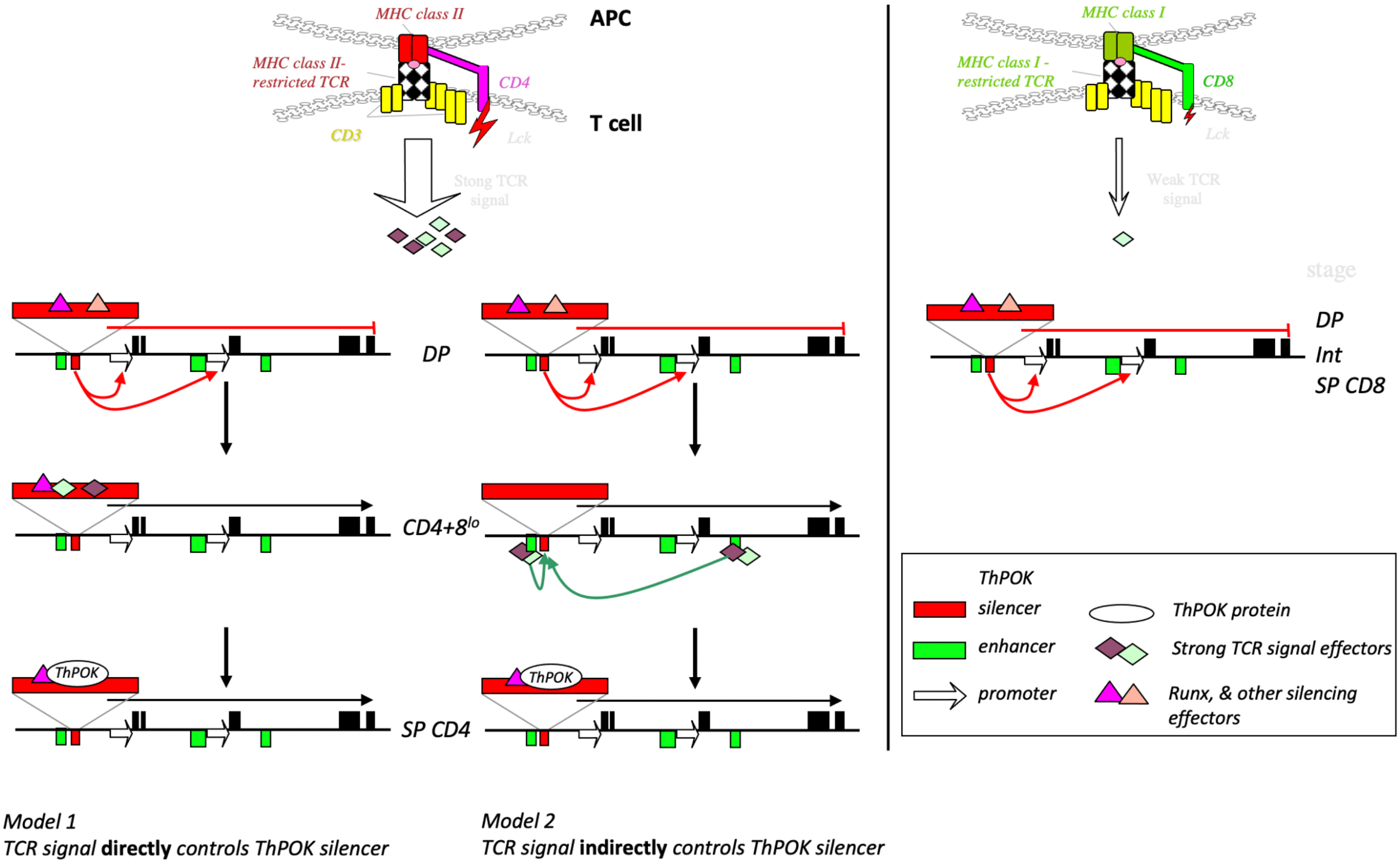

Suppl. Figure 1. Alternate models of TCR-dependent control of ThPOK transcription. Alternate models of *ThPOK* transcriptional regulation in class II-restricted thymocytes (left panel). In model 1, TCR signal strength directly controls *ThPOK* silencer function, whereas in model 2, TCR signals control activity of other cis elements, which in turn regulate silencer activity. Model of *ThPOK* transcriptional regulation in class I-restricted thymocytes (right panel).

Suppl. Fig. 2

a.

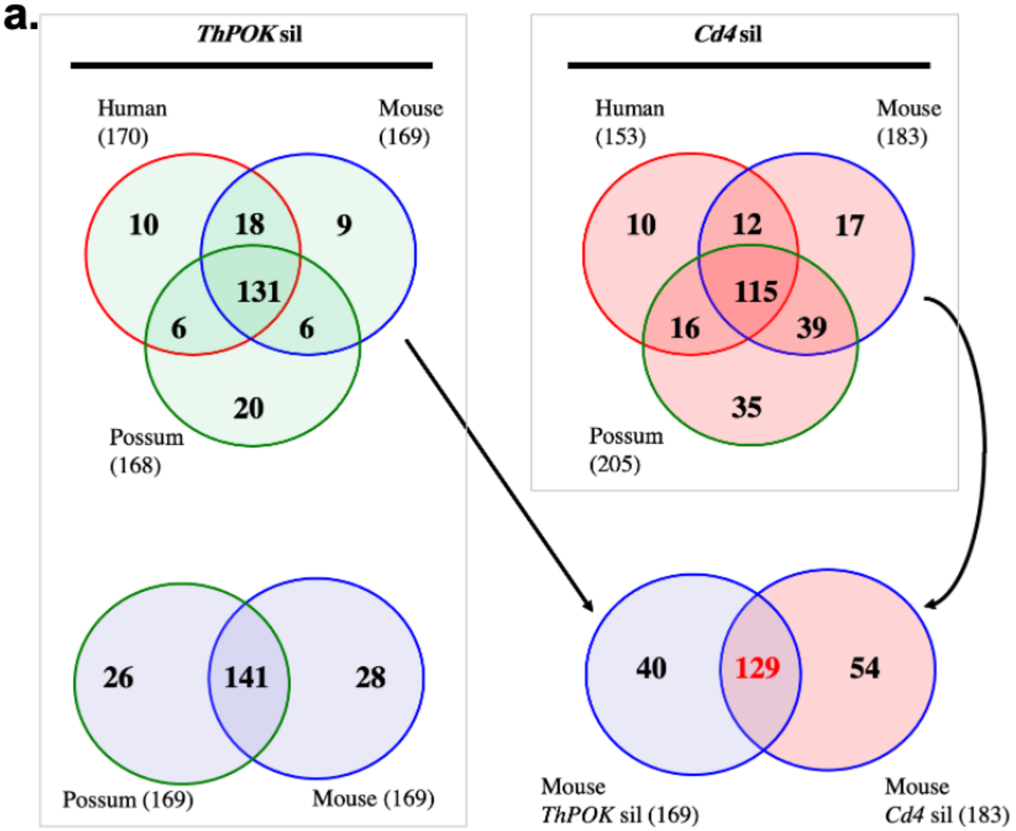

b.

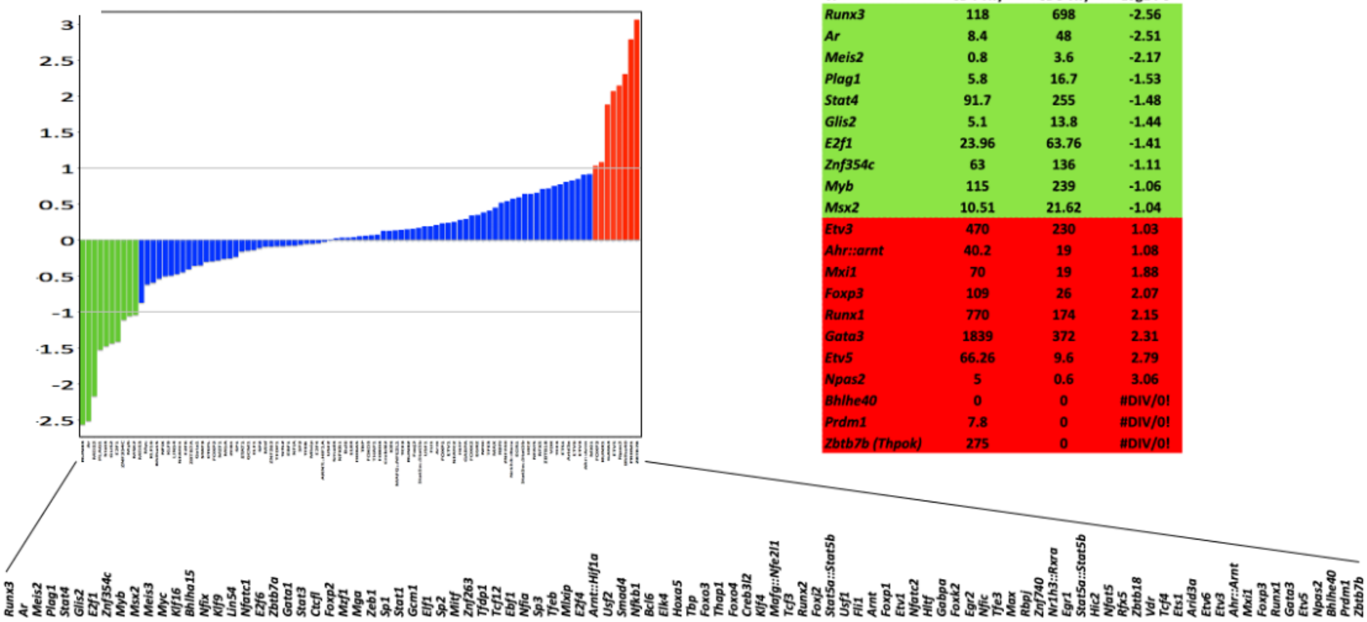

c.

TF	CD4 Thy	CD8 Thy	Log2 FC
Runx3	118	698	-2.56
Ar	8.4	48	-2.51
Meis2	0.8	3.6	-2.17
Plag1	5.8	16.7	-1.53
Stat4	91.7	255	-1.48
Glis2	5.1	13.8	-1.44
E2f1	23.96	63.76	-1.41
Znf354c	63	136	-1.11
Myb	115	239	-1.06
Msx2	10.51	21.62	-1.04
Etv3	470	230	1.03
Ahr::arnt	40.2	19	1.08
Mxi1	70	19	1.88
Foxp3	109	26	2.07
Runx1	770	174	2.15
Gata3	1839	372	2.31
Etv5	66.26	9.6	2.79
Npas2	5	0.6	3.06
Bhlhe40	0	0	#DIV/0!
Prdm1	7.8	0	#DIV/0!
Zbtb7b (Thpok)	275	0	#DIV/0!

Suppl. Figure 2. Shared TF binding sites in Sil^{ThPOK} elements, and mouse Sil^{CD4}: (a) TF consensus binding sites (as predicted by JASPAR algorithm [1], using default parameters) were mapped to *Cd4* and *ThPOK* silencers for 3 indicated species (total number of predicted consensus sites is indicated in brackets for each species). **b,c)** Focusing among these 129 TFs that are expressed in SP thymocytes, reveals a high proportion that are differentially regulated between CD4 and CD8 thymocytes and thus may be important for lineage commitment/differentiation, including Runx3 and ThPOK. Gene expression data was downloaded from IMMGEN SKYLINE RNAseq database [2], and log2 (fold change) in CD4 versus CD8 thymocytes calculated.

Suppl. Fig. 3

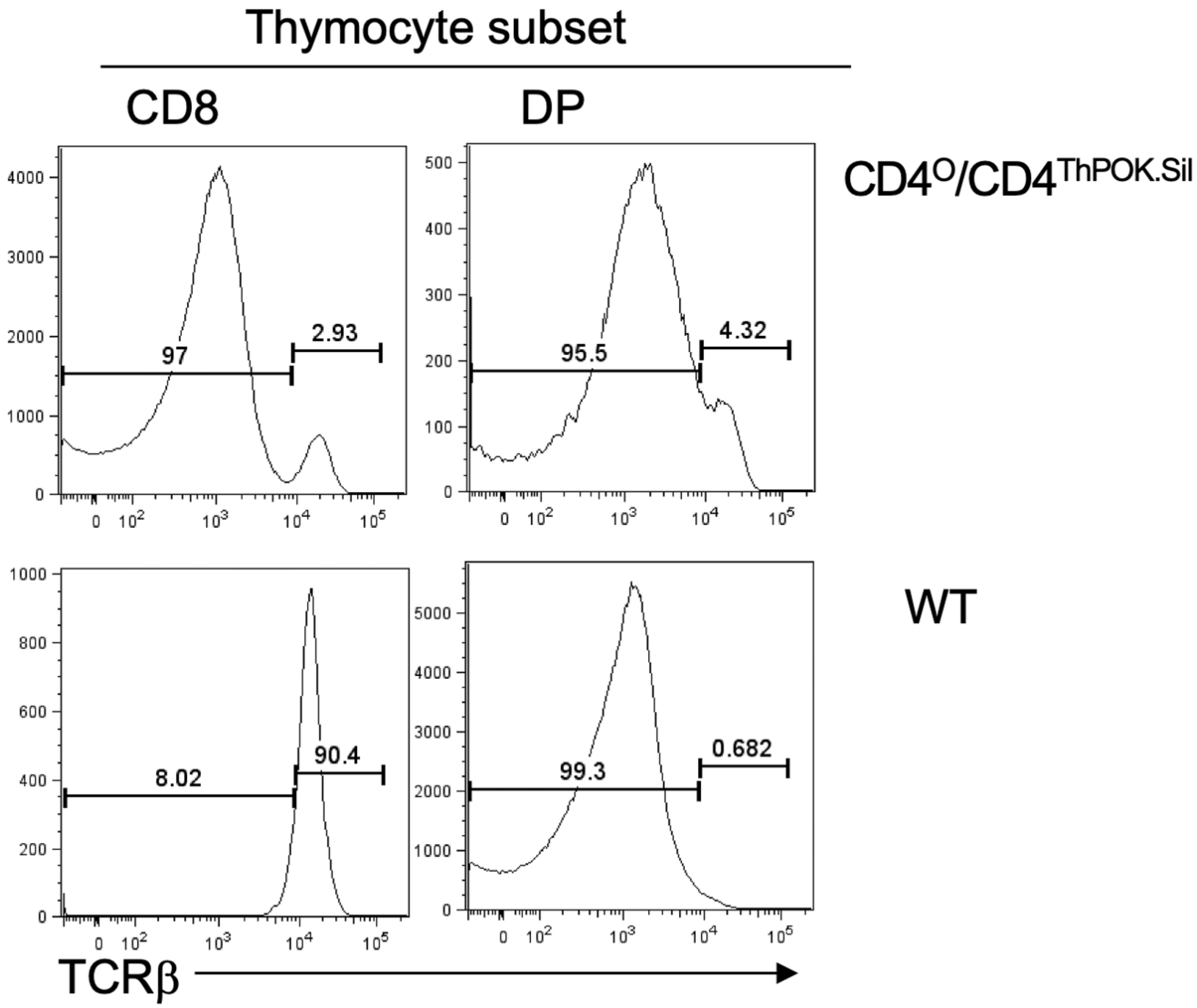

Suppl. Fig. 3. TCRβ expression of gated DP and SDP CD8 thymocytes from wt and $CD4^{ThPOK.Sil/O}$ mice, as indicated.

Suppl. Fig. 4

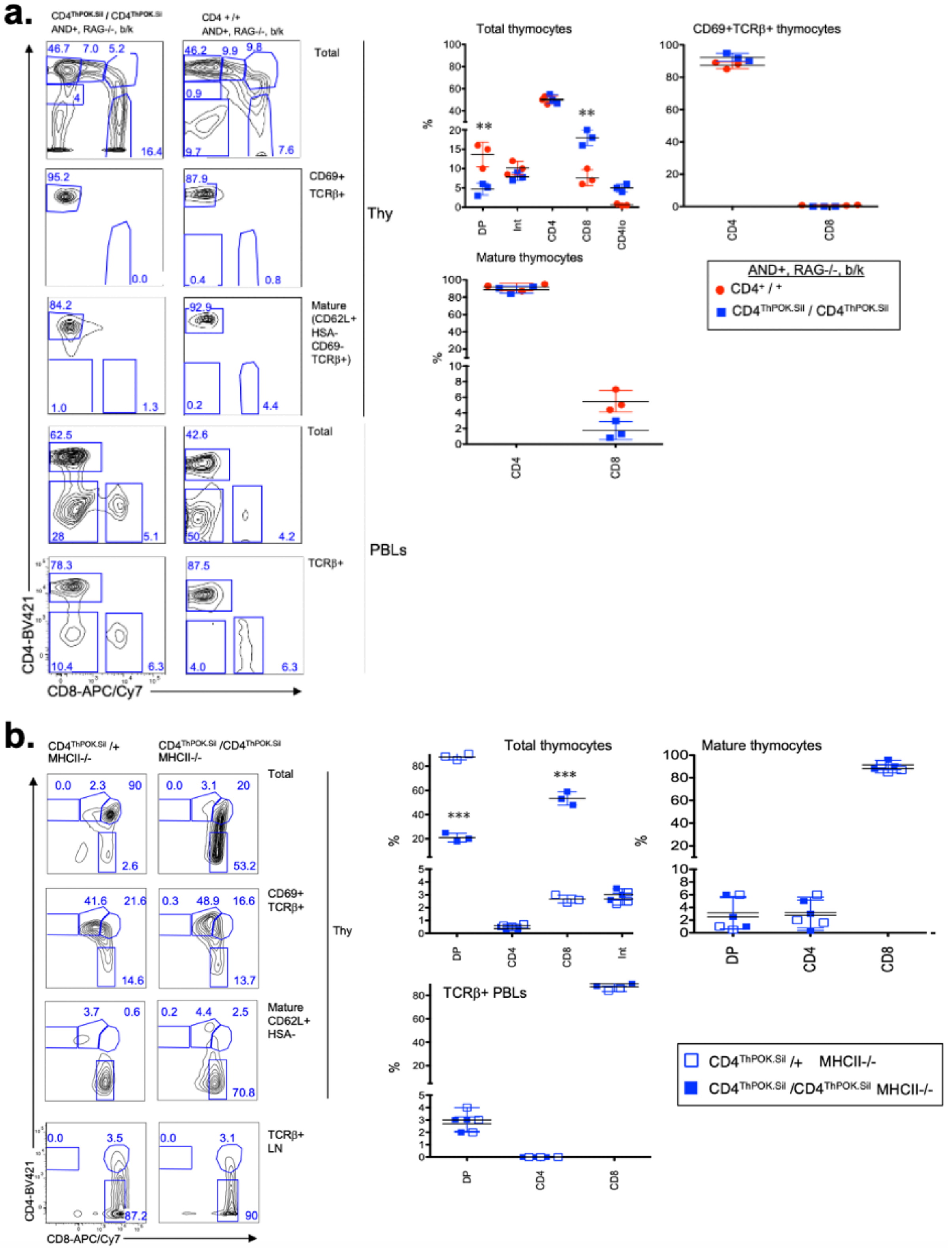

Suppl. Figure 4. CD4$^{ThPOK.Sil}$ allele supports normal development of MHC class I-restricted thymocytes, but development of class II-restricted thymocytes is impaired. FACS analysis of CD4 and CD8a expression by indicated thymic or peripheral lymphocyte populations of wt or CD4$^{ThPOK.Sil/ThPOK.Sil}$ mice back crossed **a)** AND TCR transgene on the H-2b/k background. **b)** MHCII -/- background. Plots are a combination of 3 replicates per strain, and show % of indicated populations among total thymocytes, gated TCRβ+ thymocytes, or peripheral blood lymphocytes (PBLs) for mice of each genotype. Data are presented as mean values +/- SEM. A *P* value < 0.05 was considered significant. Statistical significance was determined by one-way Anova with post-hoc Tukey HSD, and indicated by asterisks (* p < 0.01; ** p < 0.005; *** p < 0.001). Statistical significance was calculated for each indicated mutant line relative to AND+ RAG-/- H-2$^{b/k}$ CD4+/+ mice (panel a), or MHC II-/- CD4$^{ThPOK.Sil/+}$ mice (panel b).

Suppl. Fig. 5

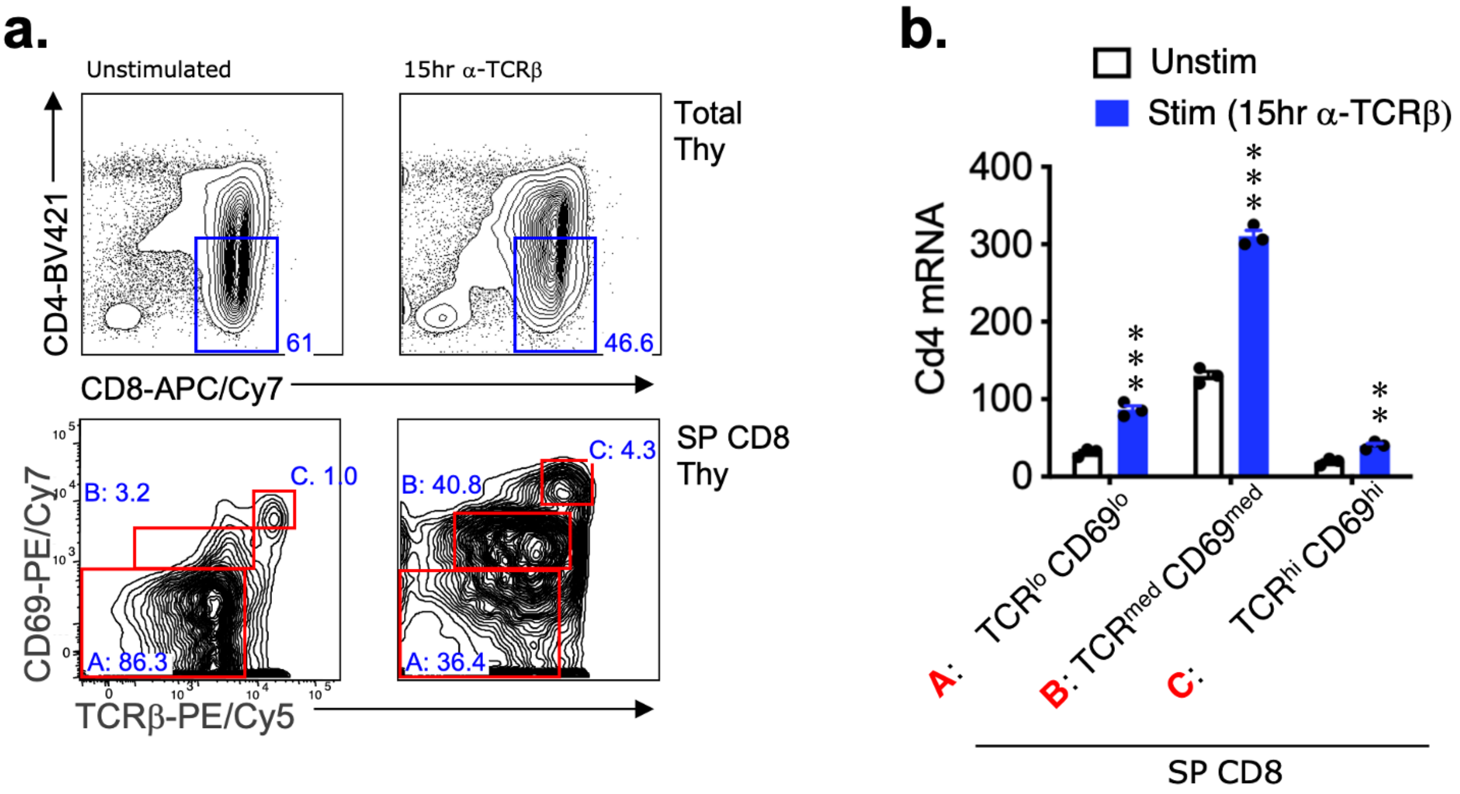

Suppl. Figure 5. Sil^{ThPOK} elements responds to TCR signal: **a)** FACS analysis of CD4, CD8a, CD69 and TCRβ expression by thymocytes of CD4$^{ThPOKsil/ThPOKsil}$ MHCII -/- mice, either injected *in vivo* with anti-TCR antibody or untreated, as indicated. Note increase in CD69+ cells indicative of recent TCR stimulation. All FACS results are representative of at least 3 experiments. **b)** RT- PCR analysis showing relative expression of *Cd4* mRNA in indicated sorted thymocyte subsets of CD4$^{ThPOKsil/ThPOKsil}$ MHCII -/- mice, either injected in vivo with anti-TCR antibody or untreated, and gated as in CD8 SP. Data were analyzed by applying unpaired two-tailed Student's t test, and one-way analysis of variance (ANOVA) with Bonferroni correction. A P value of less than 0.05 was considered significant. *P < 0.05, **P < 0.01, ***P < 0.001. All statistical analysis performed for sequencing data is mentioned elsewhere in the methods section. Data are presented as mean values +/- SEM. A P value < 0.05 was considered significant. N = 4 biological replicates. Statistical significance was calculated for each indicated stimulated cell population relative to equivalent unstimulated cell population.

Suppl. Fig. 6

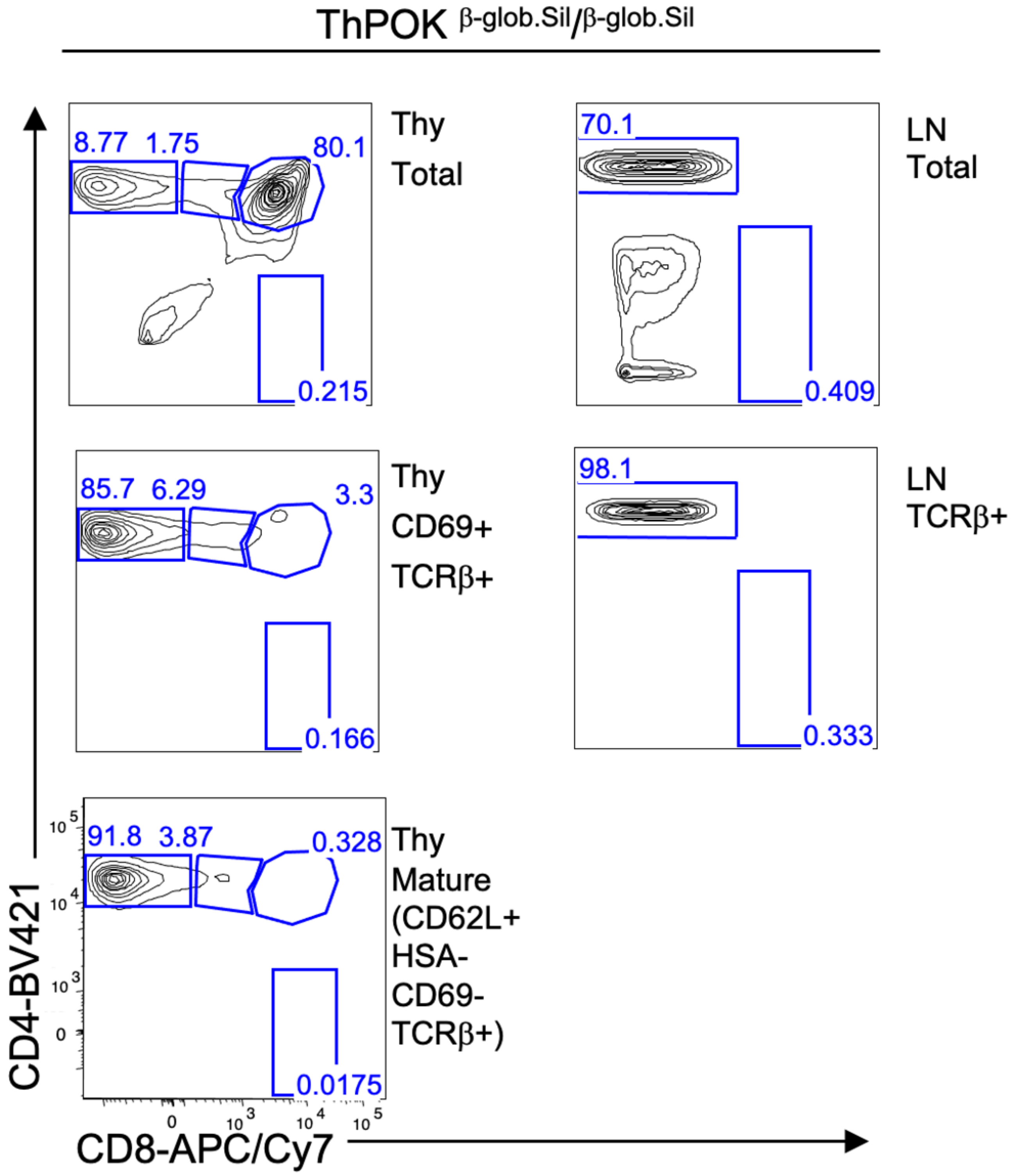

Suppl. Fig. 6. The *β-globin* silencer cannot support normal CD8 development when substituted for the endogenous *ThPOK* silencer. FACS analysis of CD4 and CD8a expression by indicated thymic or peripheral lymphocyte populations of ThPOK$^{\beta\text{- glob.Sil}/\beta\text{-glob.Sil}}$ mice. Note absence of mature SP CD8 thymocytes and SP CD8 peripheral T cells.

Suppl. Fig. 7

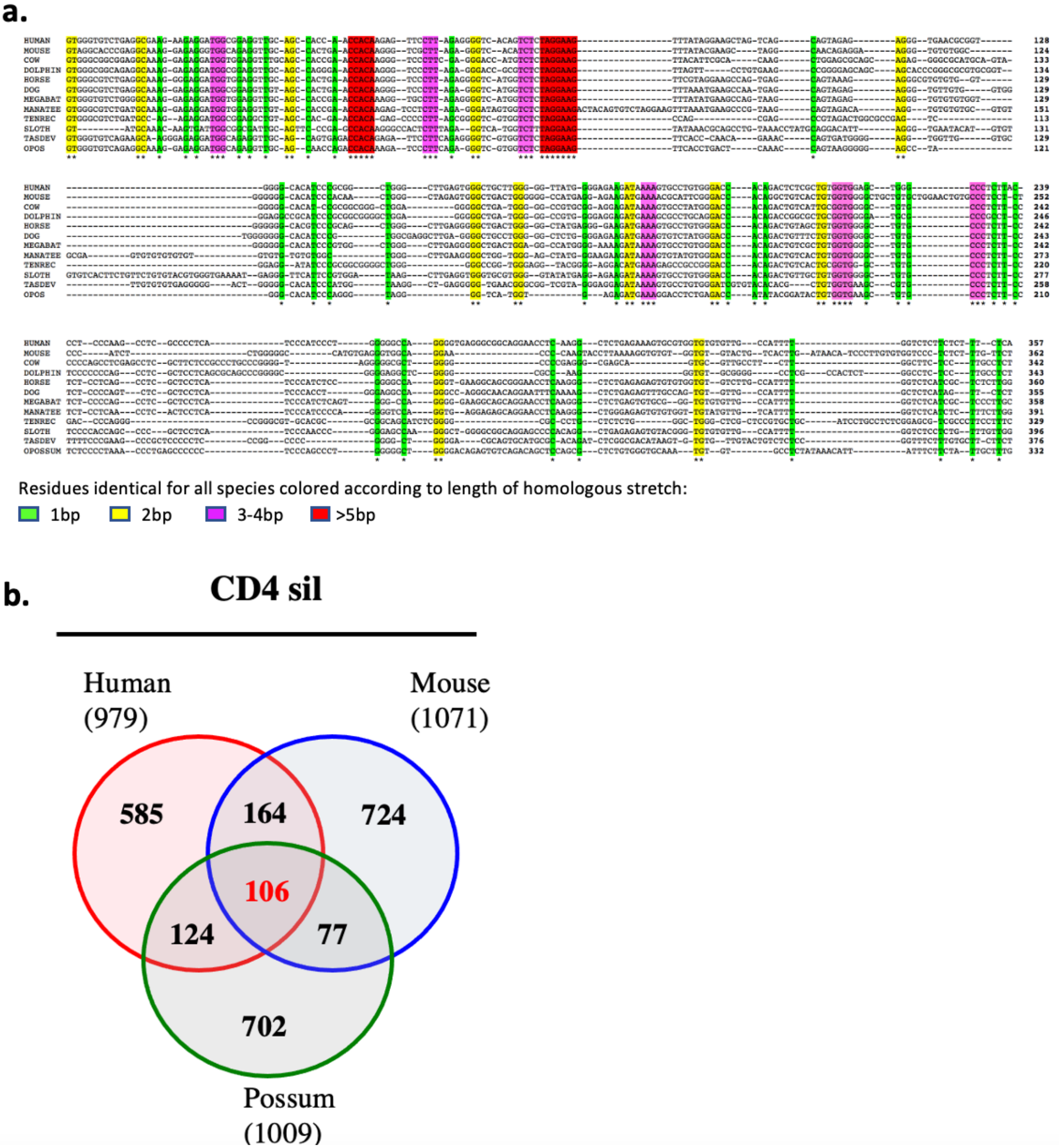

Suppl. Fig. 7. Alignments of mammalian Sil^{CD4} elements, and comparison of predicted TF binding sites between Sil^{CD4} and Sil^{ThPOK} elements. a) Alignments of *Cd4* silencers from indicated mammalian species. Residues identical for all species are colored, according to length of homologous stretch, i.e. 1bp (green), 2bp (yellow), 3-4bp (purple), >5bp (red). Note that there is very limited homology between *Cd4* silencers from different species. Comparison was carried out using Clustal Omega alignment program [3]. **b)** Venn diagram showing TF sites conserved in position and orientation between species for *Cd4* silencers of different species. TF consensus binding sites (as predicted by JASPAR algorithm) were mapped to *Cd4* silencers for 3 indicated species (total number of predicted consensus sites is indicated in brackets for each species). Using interspecies alignment, each consensus site was classified as conserved or nonconserved (in position and orientation), relative to the other species. 106 TF sites were found to be conserved in position/orientation across all 3 species for the Sil^{CD4} element.

Suppl. Fig. 8

a.

	ThPOK Hum	*ThPOK* Mouse	*ThPOK* Pos	*ThPOK* Avg	*Cd4* Hum	*Cd4* Mouse	*Cd4* Pos	*Cd4* Avg	Ratio
CENPB	2	2	2	2	0	0	0	0	#DIV/0!
Elk1	6	6	8	6.66666667	0	0	0	0	#DIV/0!
Elk3	1	1	2	1.33333333	0	0	0	0	#DIV/0!
HesX1	2	3	2	2.33333333	0	0	0	0	#DIV/0!
Mef2b	2	3	3	2.66666667	0	0	0	0	#DIV/0!
Mef2d	2	2	4	2.66666667	0	0	0	0	#DIV/0!
MIXL1	4	5	7	5.33333333	0	0	0	0	#DIV/0!
NFKB2	5	2	2	3	0	0	0	0	#DIV/0!
Prrx1	2	4	4	3.33333333	0	0	0	0	#DIV/0!
RelB	2	1	0	1	0	0	0	0	#DIV/0!
Rfx1	3	3	4	3.33333333	0	0	0	0	#DIV/0!
RFX2	2	4	3	3	0	0	0	0	#DIV/0!
RFX3	4	4	2	3.33333333	0	0	0	0	#DIV/0!
RFX4	5	4	3	4	0	0	0	0	#DIV/0!
VSX1	4	3	1	2.66666667	0	0	0	0	#DIV/0!
VSX2	2	3	1	2	0	0	0	0	#DIV/0!
MEF2A	6	8	12	8.66666667	0	0	1	0.33333333	26
Lhx4	5	7	6	6	0	1	0	0.33333333	18
E2F3	8	13	6	9	1	0	1	0.66666667	13.5
MEF2C	3	4	4	3.66666667	1	0	0	0.33333333	11
CTCFL	3	5	2	3.33333333	0	1	0	0.33333333	10
NRF1	6	5	6	5.66666667	1	0	1	0.66666667	8.5
NFAT5	2	3	1	2	0	1	0	0.33333333	6
Gabpa	6	4	10	6.66666667	1	1	2	1.33333333	5
ZNF740	24	18	23	21.6666667	1	6	6	4.33333333	5
ZBTB7B	9	5	4	6	3	1	0	1.33333333	4.5
Lhx8	4	6	3	4.33333333	2	1	0	1	4.33333333
ZNF263	17	11	15	14.3333333	5	3	2	3.33333333	4.3
REL	2	2	0	1.33333333	0	0	1	0.33333333	4
NFATC1	3	1	4	2.66666667	1	1	0	0.66666667	4
E2F4	10	12	6	9.33333333	3	2	2	2.33333333	4
NFATC2	4	5	2	3.66666667	0	2	1	1	3.66666667
ELK4	4	4	10	6	2	2	1	1.66666667	3.6
ZBTB7A	10	8	7	8.33333333	3	2	2	2.33333333	3.57142857
TFDP1	4	7	3	4.66666667	2	2	0	1.33333333	3.5
TBP	6	8	6	6.66666667	2	2	2	2	3.33333333
SP2	12	14	7	11	4	2	4	3.33333333	3.3
LHX2	1	2	0	1	0	1	0	0.33333333	3
NFATC3	1	1	1	1	1	0	0	0.33333333	3
Tcf3	16	12	14	14	4	4	6	4.66666667	3
Tcf12	7	6	7	6.66666667	1	3	3	2.33333333	2.85714286
E2F1	11	16	10	12.3333333	4	6	3	4.33333333	2.84615385
ELF1	3	2	3	2.66666667	0	1	2	1	2.66666667
KLF16	12	7	8	9	3	4	4	3.66666667	2.45454545
EGR1	20	18	20	19.3333333	11	10	5	8.66666667	2.23076923
TCF4	6	5	5	5.33333333	2	4	2	2.66666667	2

b.

	ThPOK Hum	*ThPOK* Mouse	*ThPOK* POS	*ThPOK* Avg	*Cd4* Hum	*Cd4* Mouse	*Cd4* POS	*Cd4* Avg	Ratio
**HIF1A**	0	0	0	0	1	1	1	1	0
MAFF	0	0	0	0	1	1	0	0.66666667	0
MAFK	0	0	0	0	1	1	0	0.66666667	0
**NFYB**	0	0	0	0	1	0	1	0.66666667	0
**NFYA**	0	0	0	0	4	1	2	2.33333333	0
**NR2F2**	0	0	0	0	1	1	1	1	0
**RORA,B,C**	0	0	0	0	4	5	2	3.66666667	0
**SOX13**	0	0	0	0	1	2	1	1.33333333	0
SOX15	0	0	0	0	1	1	3	1.66666667	0
Sox5	0	0	0	0	0	0	3	1	0
Sox6	0	0	0	0	1	2	2	1.66666667	0
**SOX9**	0	0	0	0	0	1	1	0.66666667	0
**Tcf7**	0	0	0	0	1	1	1	1	0
**TCF7L1/2**	0	0	0	0	2	3	3	2.66666667	0
**TFEC**	0	0	0	0	1	6	1	2.66666667	0
YY1	1	0	0	0.33333333	6	3	4	4.33333333	0.07692308
Nkx3-1	0	1	0	0.33333333	4	4	1	3	0.11111111
SREBF2	0	0	1	0.33333333	3	3	2	2.66666667	0.125
TEAD1/2/3	1	1	2	1.33333333	11	13	7	10.3333333	0.12903226
SREBF1	0	0	2	0.66666667	5	4	4	4.33333333	0.15384615
**MAFG**	1	1	1	1	4	9	6	6.33333333	0.15789474
SMAD2:3:4	0	0	1	0.33333333	1	2	3	2	0.16666667
**USF2**	1	1	1	1	2	11	4	5.66666667	0.17647059
MEIS2/3	1	2	1	1.33333333	2	9	10	7	0.19047619
**NR2F1**	0	0	1	0.33333333	1	2	2	1.66666667	0.2
Pparg::Rxra	0	0	2	0.66666667	1	4	5	3.33333333	0.2
**Mitf**	1	1	1	1	2	10	3	5	0.2
Rxra	1	0	1	0.66666667	2	1	6	3	0.22222222
**STAT1**	1	2	0	1	7	4	2	4.33333333	0.23076923
Smad4	0	1	1	0.66666667	2	3	3	2.66666667	0.25
**TFE3**	2	2	1	1.66666667	4	13	3	6.66666667	0.25
NR4A2	1	0	1	0.66666667	1	3	3	2.33333333	0.28571429
PRDM1	0	1	1	0.66666667	3	3	1	2.33333333	0.28571429
**Stat4**	1	1	1	1	5	3	2	3.33333333	0.3
HLTF	1	2	3	2	3	10	5	6	0.33333333
Tcfl5	2	4	0	2	8	8	2	6	0.33333333

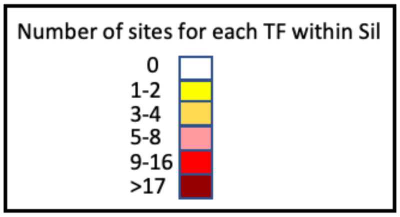

c.

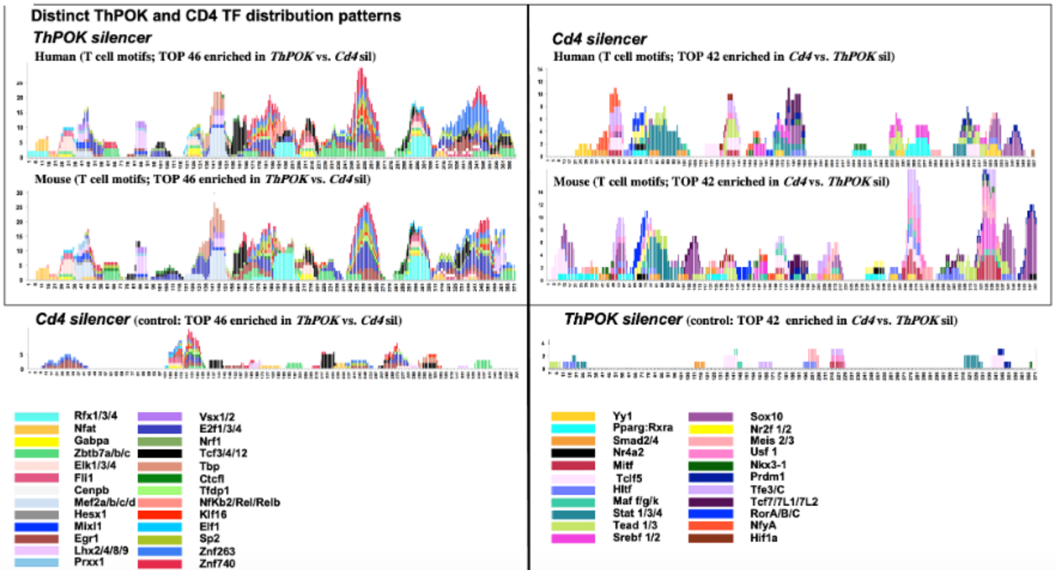

Suppl. Fig. 8: Heat map showing number of binding sites for TFs predicted to recognize the Sil^{ThPOK} and/or Sil^{CD4}. Number of sites for each TF is indicated by different colors: 0 = white; 1-2 = yellow; 3-4 = orange; 5-8 = Pink; 9-16 = red; >17 = dark maroon. TF sites are filtered for recognition by TFs that are expressed in T cells (according to IMMGEN Skyline RNAseq database), and for sites that are conserved between human and mouse homologs of either Sil^{ThPOK} and Sil^{CD4} (or both). After applying these filters, consensus sites for 172 different TFs that remained were arranged according to relative abundance in *ThPOK* versus *Cd4* silencer (ratio of # sites per silencer). **a)** Top 46 selectively enriched sites in Sil^{ThPOK}. Note enrichment of NfkB, NFAT, Egr and E box sites (Tcf3 = E2A, Tcf4 = E2-2, Tcf12= HEB), representing putative TCR regulatory signature. **b)** Top 42 selectively enriched sites in Sil^{CD4}. (c) Positions of TF consensus motifs enriched for each silencer were mapped onto the indicated full-length mouse and human silencers. Height of the peak at each base pair position corresponds to the number of overlapping TF consensus motifs at that position. Note that factors with related consensus sites were combined into groups of same color for easier visualization. Color codes for left and right sides of the diagram are different.

Suppl. Fig. 9

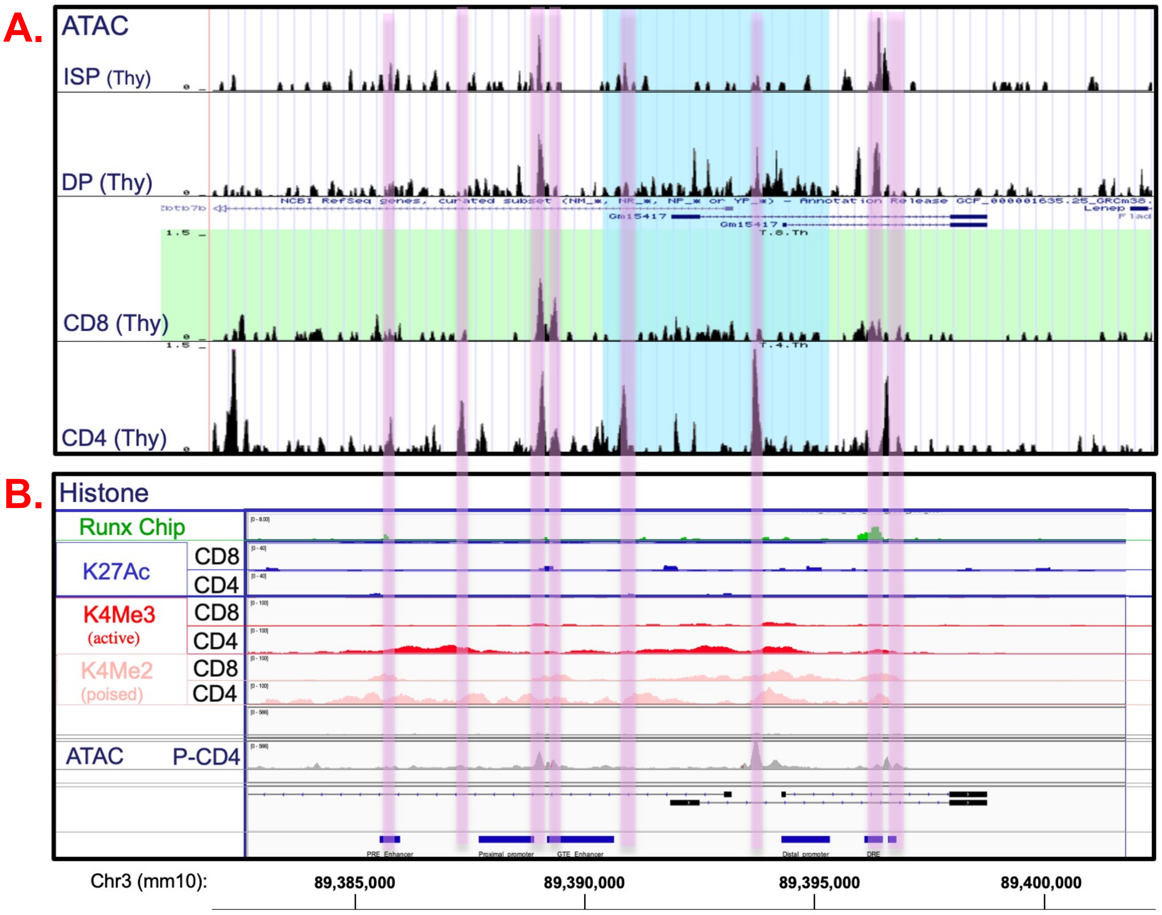

Suppl. Fig. 9. Chromatin accessibility of mouse *ThPOK* locus. A) ATAC seq B) Chip seq track of *ThPOK* locus: regulatory elements are marked with yellow bar. Note that distal Promoter (DP) part of GTE enhancer only accessible in CD4 thymocytes and peripheral CD4 (P-CD4). *ThPOK* silencer is accessible in ISP and DP, but not in CD4 or CD8 thymocytes.

Suppl. Fig. 10

Gating strategy for immature signaled and mature thymocytes

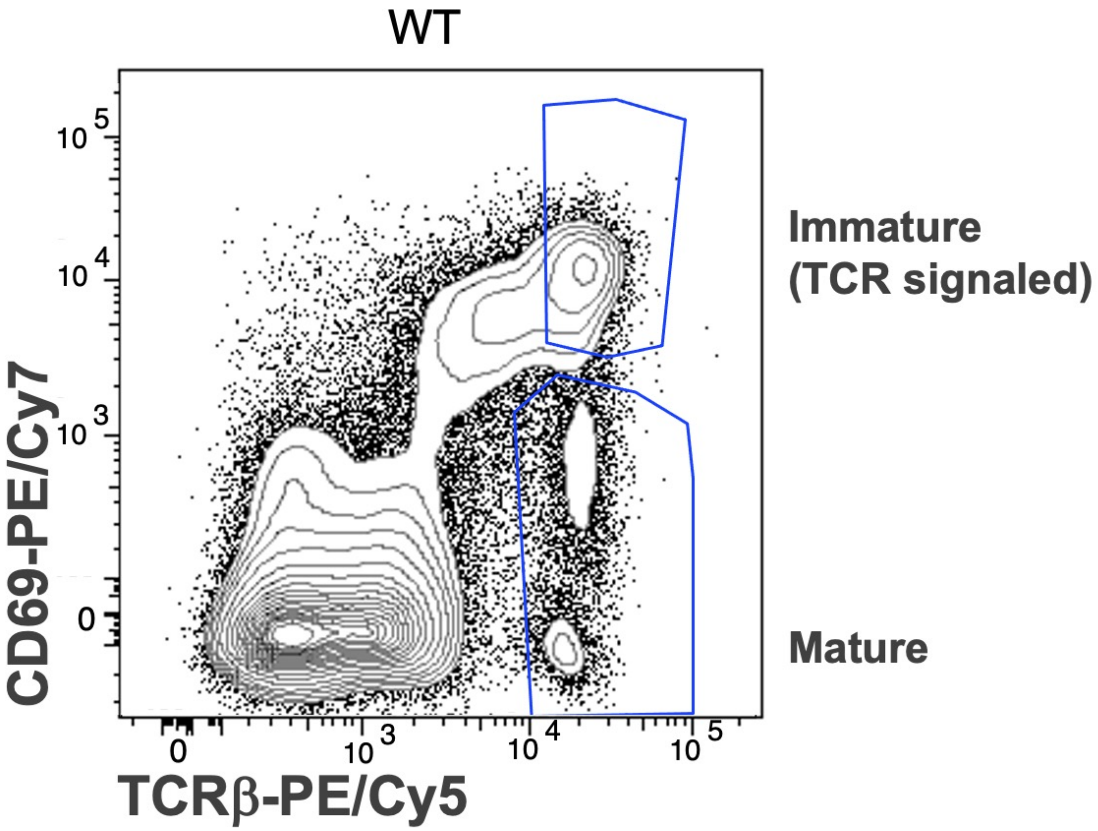

Suppl. Fig. 10. Gating strategy for immature signaled (TCRβ+ CD69+) and mature (TCRβ+ CD69-) thymocytes.

Suppl. Fig. 11

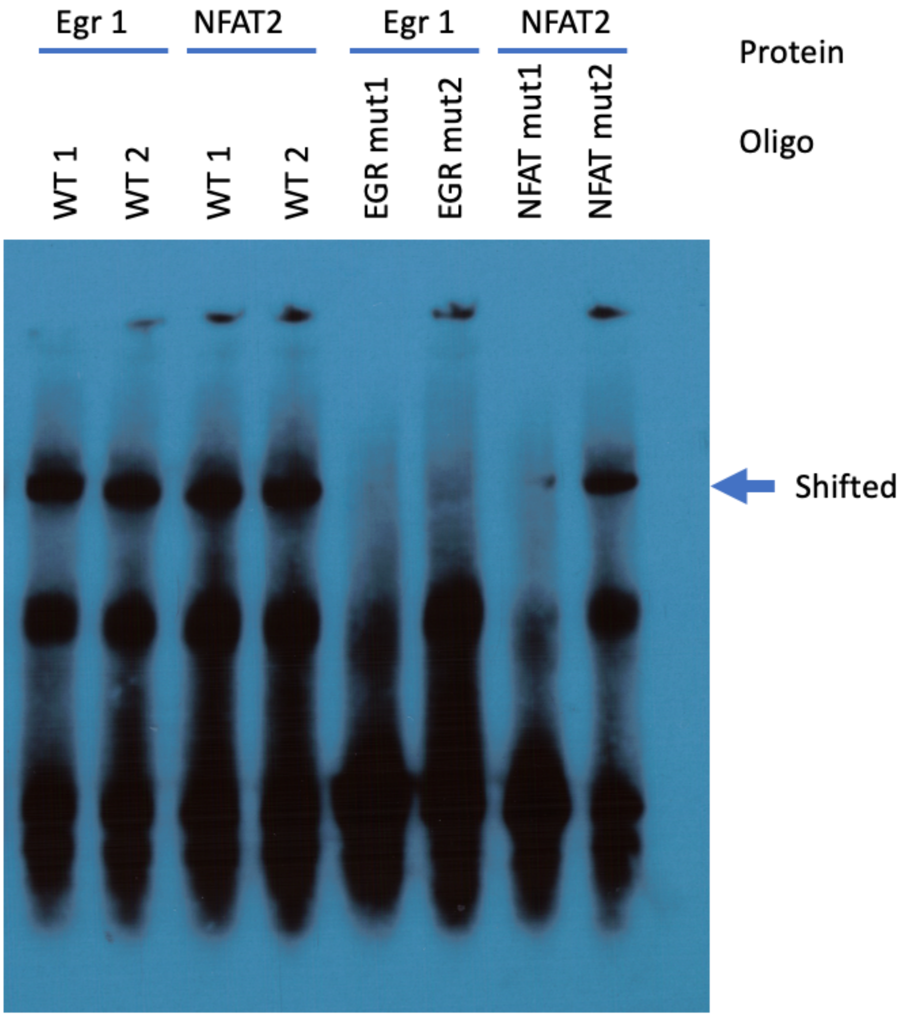

Suppl. Fig. 11. Uncropped and unedited blot/gel images corresponding to Fig. 7a**.**

Suppl. Fig. 12

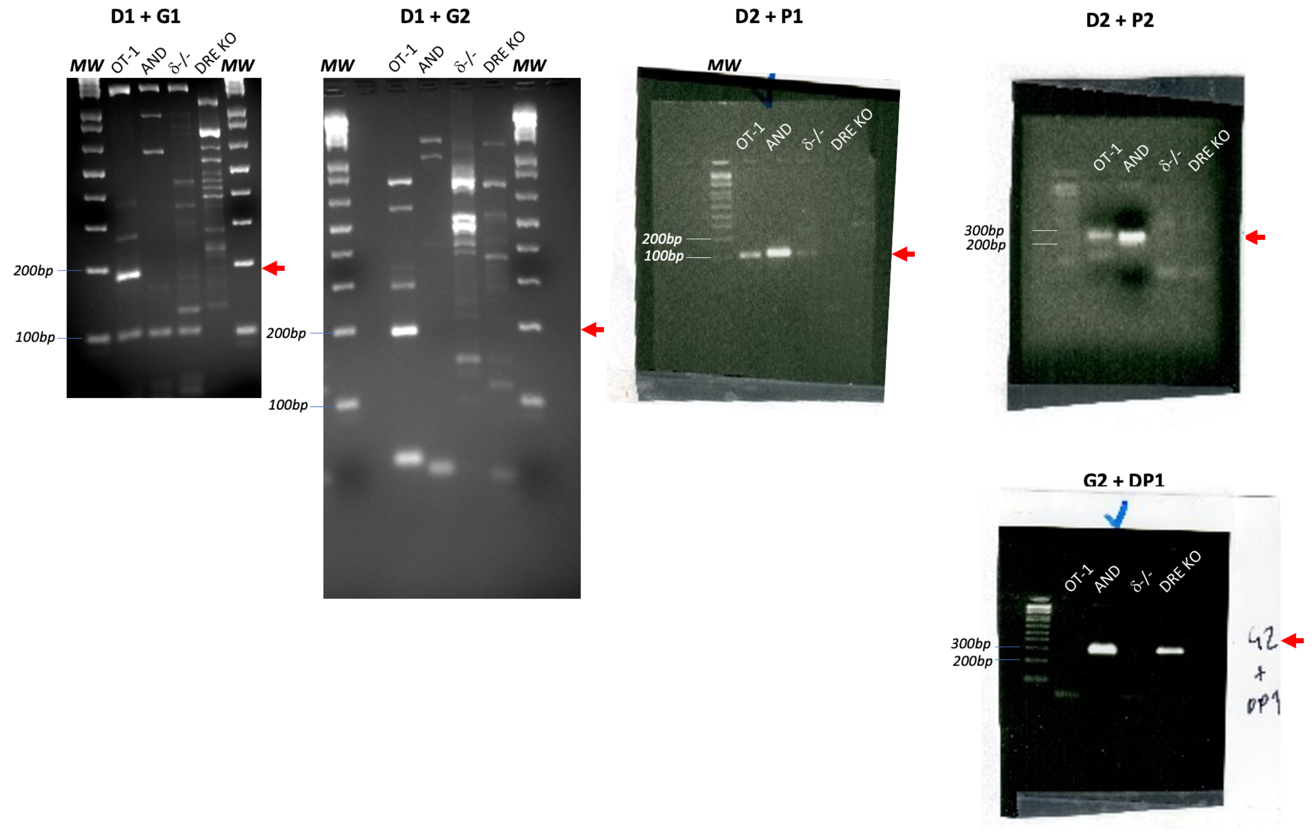

Suppl. Fig. 12. Uncropped and unedited blot/gel images corresponding to Fig. 10b**.**

Supplementary References:

1. RNAseq data sets. *Immunological Genome Consortium.* http://rstats.immgen.org/Skyline/skyline.html (2021).

2. Clustal Omega tool. *The EMBL-EBI bioinformatics web and programmatic tools framework.* https://www.ebi.ac.uk/Tools/msa/clustalo/ (2021).

3. Fornes O, Castro-Mondragon JA, Khan A, et al. *JASPAR 2020: update of the open-access database of transcription factor binding profiles*. http://jaspar.genereg.net (2021).
