# Supplementary material for: An autonomous TCR signal-sensing switch influences CD4/CD8 lineage choice in mice
Source: Commun Biol. 2022 Jan 21;5:84. doi: 10.1038/s42003-022-02999-5 (PMC8783009; doi:10.1038/s42003-022-02999-5)
Supplement: Supplementary file 2 — Description of Additional Supplementary Files [file 42003_2022_2999_MOESM2_ESM.pdf]

Description of Additional Supplementary Files

File name: Supplementary Data 1.
Description: Source data for Figure graphs.
