# Supplementary material for: An autonomous TCR signal-sensing switch influences CD4/CD8 lineage choice in mice
Source: Commun Biol. 2022 Jan 21;5:84. doi: 10.1038/s42003-022-02999-5 (PMC8783009; doi:10.1038/s42003-022-02999-5)
Supplement: Supplementary file 4 — Reporting Summary [file 42003_2022_2999_MOESM4_ESM.pdf]

nature research

Corresponding author(s): Dietmar Kappes
Last updated by author(s): 10-19-21

# Reporting Summary

Nature Research wishes to improve the reproducibility of the work that we publish. This form provides structure for consistency and transparency in reporting. For further information on Nature Research policies, see our Editorial Policies and the Editorial Policy Checklist.

## Statistics

For all statistical analyses, confirm that the following items are present in the figure legend, table legend, main text, or Methods section.

| n/a | Confirmed | |
|---|---|---|
| ☐ | ☒ | The exact sample size (*n*) for each experimental group/condition, given as a discrete number and unit of measurement |
| ☐ | ☒ | A statement on whether measurements were taken from distinct samples or whether the same sample was measured repeatedly |
| ☐ | ☒ | The statistical test(s) used AND whether they are one- or two-sided<br>*Only common tests should be described solely by name; describe more complex techniques in the Methods section.* |
| ☐ | ☒ | A description of all covariates tested |
| ☐ | ☒ | A description of any assumptions or corrections, such as tests of normality and adjustment for multiple comparisons |
| ☐ | ☒ | A full description of the statistical parameters including central tendency (e.g. means) or other basic estimates (e.g. regression coefficient) AND variation (e.g. standard deviation) or associated estimates of uncertainty (e.g. confidence intervals) |
| ☐ | ☒ | For null hypothesis testing, the test statistic (e.g. *F*, *t*, *r*) with confidence intervals, effect sizes, degrees of freedom and *P* value noted<br>*Give P values as exact values whenever suitable.* |
| ☒ | ☐ | For Bayesian analysis, information on the choice of priors and Markov chain Monte Carlo settings |
| ☒ | ☐ | For hierarchical and complex designs, identification of the appropriate level for tests and full reporting of outcomes |
| ☒ | ☐ | Estimates of effect sizes (e.g. Cohen's *d*, Pearson's *r*), indicating how they were calculated |

*Our web collection on statistics for biologists contains articles on many of the points above.*

## Software and code

Policy information about availability of computer code

| | |
|---|---|
| Data collection | na |
| Data analysis | na |

For manuscripts utilizing custom algorithms or software that are central to the research but not yet described in published literature, software must be made available to editors and reviewers. We strongly encourage code deposition in a community repository (e.g. GitHub). See the Nature Research guidelines for submitting code & software for further information.

## Data

Policy information about availability of data

All manuscripts must include a data availability statement. This statement should provide the following information, where applicable:

- Accession codes, unique identifiers, or web links for publicly available datasets
- A list of figures that have associated raw data
- A description of any restrictions on data availability

na

# Field-specific reporting

Please select the one below that is the best fit for your research. If you are not sure, read the appropriate sections before making your selection.

☒ Life sciences ☐ Behavioural & social sciences ☐ Ecological, evolutionary & environmental sciences

For a reference copy of the document with all sections, see nature.com/documents/nr-reporting-summary-flat.pdf

# Life sciences study design

All studies must disclose on these points even when the disclosure is negative.

| | |
|---|---|
| Sample size | No statistical methods were used to predetermine sample size. Instead, sample sizes were rationalized by weighing sufficient replication (to determine the extent of biological variation) with reduction of total animals used. |
| Data exclusions | No biological data was excluded. |
| Replication | Results were confirmed by analysis of individual biological replicates, and all attempts at replication were successful. |
| Randomization | Sample allocation was not random. Instead, biological controls were included in all experiments. |
| Blinding | Blinding was not possible, since genotyping was necessary for all mouse experiments. |

# Reporting for specific materials, systems and methods

We require information from authors about some types of materials, experimental systems and methods used in many studies. Here, indicate whether each material, system or method listed is relevant to your study. If you are not sure if a list item applies to your research, read the appropriate section before selecting a response.

## Materials & experimental systems

| n/a | Involved in the study | |
|---|---|---|
| ☐ | ☒ | Antibodies |
| ☐ | ☒ | Eukaryotic cell lines |
| ☒ | ☐ | Palaeontology and archaeology |
| ☐ | ☒ | Animals and other organisms |
| ☒ | ☐ | Human research participants |
| ☒ | ☐ | Clinical data |
| ☒ | ☐ | Dual use research of concern |

## Methods

| n/a | Involved in the study | |
|---|---|---|
| ☒ | ☐ | ChIP-seq |
| ☐ | ☒ | Flow cytometry |
| ☒ | ☐ | MRI-based neuroimaging |

## Antibodies

| | |
|---|---|
| Antibodies used | All fluorescently labeled antibodies used were obtained from commercial sources (eBioscience, Biolegend, BD, or Invitrogen), including TCRβ-PE/Cy5 (H57-597), CD4-BV421 (RM4-5), CD8a-APC/Cy7 (53-6.7), CD69-PE/Cy7 (H1.2F3), CD24-FITC (M1-69), CD62L-PerCP/Cy5.5 (MEL-14). |
| Validation | All antibodies were validated by the source company. |

## Eukaryotic cell lines

Policy information about cell lines

| | |
|---|---|
| Cell line source(s) | HEK 293T |
| Authentication | Authenticity verified by supplier (ATCC ), according to product information sheet. |
| Mycoplasma contamination | HEK293T from this source have been confirmed as negative for mycoplasma. |
| Commonly misidentified lines (See ICLAC register) | na |

## Animals and other organisms

Policy information about studies involving animals; ARRIVE guidelines recommended for reporting animal research

| | |
|---|---|
| Laboratory animals | Male and female mice were maintained on a C57BL/6 background and were analyzed between 5 and 12 weeks of age. |
| Wild animals | The study did not involve samples collected from wild animals. |
| Field-collected samples | The study did not involve samples collected from the field |
| Ethics oversight | All experimentation involving animals was approved by Institutional Animal Care and Use Committee (IACUC), of Fox Chase Cancer Center. |

Note that full information on the approval of the study protocol must also be provided in the manuscript.

## Flow Cytometry

### Plots

Confirm that:

☒ The axis labels state the marker and fluorochrome used (e.g. CD4-FITC).

☒ The axis scales are clearly visible. Include numbers along axes only for bottom left plot of group (a 'group' is an analysis of identical markers).

☐ All plots are contour plots with outliers or pseudocolor plots.

☒ A numerical value for number of cells or percentage (with statistics) is provided.

### Methodology

| | |
|---|---|
| Sample preparation | Mice were euthanized using carbon dioxide followed by cervical dislocation. Mesenteric lymph nodes, thymus and spleen were harvested immediately after euthanasia and stored in cold medium (2% FBS, RPMI) under sterile conditions. Single-cell sell suspensions were obtained by crushing organs through a 40 µm cell strainer (Becton, Dickinson and Company). Prior to analytical flow cytometry, spleen and lymph node samples were layered over Lympholyte-M (Cedarlane), centrifuged at 1,200rpm for 30', and interface collected. |
| Instrument | Flow cytometry analyses were conducted on a FACS LSRII or FACS LSRFortessa (Becton, Dickinson, and Company). Cell sorting was performed on a FACSAria II (Becton, Dickenson, and Company). |
| Software | FACS data was analyzed using FlowJo software (version 10.1 or 10.2, FlowJo, Ashland, OR, USA). |
| Cell population abundance | No post-sort analysis was performed. |
| Gating strategy | For all analyses, PI+ or 7AAD+ cells were excluded, then debris was excluded using a FSC-A vs SSC-A gate, then doublets were excluded using FSC-W vs FSC-H or SSC-W vs SSC-H gates for all downstream gating. Fluorescent minus-one controls were used in some circumstances to assist in discriminating between positive and negative signal, while other gating was performed according to previously published strategies. |

☒ Tick this box to confirm that a figure exemplifying the gating strategy is provided in the Supplementary Information.
